# The Impact of COVID-19 on Mortality in Italy: Retrospective Analysis of Epidemiological Trends

**DOI:** 10.2196/36022

**Published:** 2022-04-07

**Authors:** Alessandro Rovetta, Akshaya Srikanth Bhagavathula

**Affiliations:** 1 R & C Research Bovezzo (Brescia) Italy; 2 Institute of Public Health College of Medicine and Health Sciences United Arab Emirates University Abu Dhabi United Arab Emirates

**Keywords:** COVID-19, deniers, excess deaths, epidemiology, infodemic, infodemiology, Italy, longitudinal analysis, mortality, time series, pandemic, public health

## Abstract

**Background:**

Despite the available evidence on its severity, COVID-19 has often been compared with seasonal flu by some conspirators and even scientists. Various public discussions arose about the noncausal correlation between COVID-19 and the observed deaths during the pandemic period in Italy.

**Objective:**

This paper aimed to search for endogenous reasons for the mortality increase recorded in Italy during 2020 to test this controversial hypothesis. Furthermore, we provide a framework for epidemiological analyses of time series.

**Methods:**

We analyzed deaths by age, sex, region, and cause of death in Italy from 2011 to 2019. Ordinary least squares (OLS) linear regression analyses and autoregressive integrated moving average (ARIMA) were used to predict the best value for 2020. A Grubbs 1-sided test was used to assess the significance of the difference between predicted and observed 2020 deaths/mortality. Finally, a 1-sample *t* test was used to compare the population of regional excess deaths to a null mean. The relationship between mortality and predictive variables was assessed using OLS multiple regression models. Since there is no uniform opinion on multicomparison adjustment and false negatives imply great epidemiological risk, the less-conservative Siegel approach and more-conservative Holm-Bonferroni approach were employed. By doing so, we provided the reader with the means to carry out an independent analysis.

**Results:**

Both ARIMA and OLS linear regression models predicted the number of deaths in Italy during 2020 to be between 640,000 and 660,000 (range of 95% CIs: 620,000-695,000) against the observed value of above 750,000. We found strong evidence supporting that the death increase in all regions (average excess=12.2%) was not due to chance (t_21_=7.2; adjusted *P*<.001). Male and female national mortality excesses were 18.4% (*P*<.001; adjusted *P*=.006) and 14.1% (*P*=.005; adjusted *P*=.12), respectively. However, we found limited significance when comparing male and female mortality residuals’ using the Mann-Whitney *U* test (*P*=.27; adjusted *P*=.99). Finally, mortality was strongly and positively correlated with latitude (*R*=0.82; adjusted *P*<.001). In this regard, the significance of the mortality increases during 2020 varied greatly from region to region. Lombardy recorded the highest mortality increase (38% for men, adjusted *P*<.001; 31% for women, *P*<.001; adjusted *P*=.006).

**Conclusions:**

Our findings support the absence of historical endogenous reasons capable of justifying the mortality increase observed in Italy during 2020. Together with the current knowledge on SARS-CoV-2, these results provide decisive evidence on the devastating impact of COVID-19. We suggest that this research be leveraged by government, health, and information authorities to furnish proof against conspiracy hypotheses that minimize COVID-19–related risks. Finally, given the marked concordance between ARIMA and OLS regression, we suggest that these models be exploited for public health surveillance. Specifically, meaningful information can be deduced by comparing predicted and observed epidemiological trends.

## Introduction

### Background

SARS-CoV-2 is a new beta coronavirus first identified in December 2019 in Wuhan, China. The related pathology, called COVID-19, has raged worldwide, claiming millions of victims and throwing economic and health systems into severe crises. In such a dramatic scenario, Europe is one of the most affected areas: As of December 2021, it accounted for over 30% of global official deaths (ie, approximately 1,600,000) [1]. Because the risk factors are multiple, including environmental conditions, pollution, age, gender, ethnicity, crowding, poverty, and medical comorbidities, mortality varies substantially from country to country as well as intranationally [2-4]. Indeed, the peaks in daily deaths per million inhabitants ranged from 1 (Ukraine) to over 40 (Belgium), with a median of 3.5 (IQR 2-13) [4]. The first European nation to suffer the devastating effects of COVID-19 was Italy, with mortality peaks much higher than the European median (over 15). In particular, the regions of northern Italy—especially the provinces of Bergamo and Brescia—faced a harsh first wave, reaching the highest number of deaths globally [1,5]. To date, despite a substantial reduction in mortality thanks to a massive vaccination campaign, Italy is still the second-ranking European country for official COVID-19 deaths [1,6]. Nonetheless, the debate over COVID-19 mortality has been intense during the pandemic. In the early stages, given the low testing capabilities, the calculation of mortality was subject to numerous uncertainties, which led to both overestimates and underestimates. For this reason, researchers focused their efforts on comparing 2020 data with historical death series [7].

Ordinary least squares (OLS) regression models are among the most adopted model by scientists due to their simplicity and efficacy. Specifically, OLS multiple and simple regressions have often been used to predict the course of COVID-19 cases and deaths, both individually and in conjunction with other epidemiological models such as Susceptible-Infected-Recovered (SIR) [8-10]. The literature shows that linear regression analyses are valuable short-term forecasting tools when the necessary assumptions are satisfied. However, it is not unusual for requirements such as normality of the residuals or homoskedasticity to be violated when dealing with actual epidemiological data. In these cases, the use of corrective procedures or alternative models should be considered. Among the latter, autoregressive integrated moving average (ARIMA) and SARIMA (ARIMA + seasonal component) models have shown excellent predictive capabilities. In particular, a recent study by Abolmaali and Shirzaei [11] demonstrated that the ARIMA approach could outperform other classical models such as logistic function, linear regression, and SIR. Similar findings were obtained by Alabdulrazzaq et al [12], who proved that the accuracy of the prediction of COVID-19 spread provided by their ARIMA model was both appropriate and satisfactory.

Despite more than 135,000 official deaths nationwide, some Italian conspiracy movements argue that COVID-19 is a nondangerous disease and that these numbers have been deliberately exaggerated [13]. Unfortunately, it was not uncommon even for eminent Italian scientists or other prominent personalities to have recklessly downplayed the risks of COVID-19 or favored the spread of fake news [14,15]. Thus, the infodemic question “Dead from COVID or with COVID?” soon filled social networks [13]. Indeed, such a question arises from the hypothesis that COVID-19 was noncausally correlated with the deaths recorded in Italy in 2020.

### Objective

Based on this premise, this study aimed to estimate the difference between the observed and predicted numbers of deaths in Italy during 2020. In particular, we modelled all mortality trends by cause of death, sex, and age group from 2011 to 2019, predicting the best values for 2020. By doing so, causal evidence will be provided on the impact of a nonendogenous mortality factor, such as COVID-19. The results of this paper have epidemiological and infodemiological relevance since (1) 2 models widely adopted by the scientific community such as OLS linear regression and ARIMA are compared; (2) to the best of our knowledge, this is the most detailed historical and forecasting survey regarding mortality in Italy; and (3) an estimate of the statistical significance of the increase in mortality in Italy during 2020 is provided. Finally, we investigate 2 essential, but often overlooked, aspects of epidemiological and public health surveillance, namely the possible emergence of nonlinear subtrends (capable of invalidating predictions of models trained on historical global data) and the problem of multicomparison adjustment (capable of dangerously inflating false negatives).

## Methods

### Data Collection

For this study, we used data from the national agencies and portals of demographic and statistical research, Italy (details and references are provided in the following paragraphs). Specifically, the annual number of deaths (including deaths by sex and age groups), deaths per causes of deaths (including deaths by sex groups), and mortality (including mortality by sex and age groups) were extracted from the platforms and annual reports of the National Institute of Statistics (ISTAT) and National Health Observatory for the years 2011 to 2020 [16-18]. Demographic data (ie, population number per age group, population number and density, and per region) were gathered from Tuttitalia.it [19,20]. This portal contains all ISTAT demographic information relating to municipalities, provinces, and regions. Although the investigated period ranged from 2011 to 2020, causes of death statistics were available until 2017 as the official evaluation process takes 3 years [17]. More details on the data collection process are described in Multimedia Appendix 1.

### Procedure and Statistical Analysis Key Points

Here, we provide a summary of the procedure adopted. A more detailed description is reported in Multimedia Appendix 1. We modeled regional trends in annual deaths and mortality from 2011 to 2019 through OLS linear regression. We called Δ* the residuals’ data set from 2011 to 2019 and Δ the residuals’ data set from 2011 to 2020. Through the Grubbs 1-sided test, we searched for high outliers in Δ* and Δ. The Grubbs test was performed using RStudio v.4.1.2 software (library: outliers). We also performed a 1-sample *t* test to assess if the regional death increases were due to chance. This was done by comparing the 2020 excess death population to a fixed null mean (ie, the expected residual). Furthermore, we calculated the difference between the model prediction and the observed value. To validate or deny any statistical anomalies in the number of deaths during 2020, we checked all the trends of the following annual statistics within the 2011-2019 time frame: male deaths by age group, female deaths by age group, male mortality by age group, female mortality by age group, deaths by causes of death, male deaths by causes of death, female deaths by causes of death. Specifically, we searched for anomalous nonlinear subtrends capable of distorting the interpretations on the cumulative data (indeed, sum of linear trends is linear). An example of this phenomenon is shown in Figure S1 in Multimedia Appendix 1. Concerning male and female deaths for age groups, we also calculated the 2020 forecast for each age group through an ARIMA (p, d, q) model using RStudio v.4.1.2 software (libraries: forecast and tseries). To facilitate the reproducibility of the analysis, we have provided all the ARIMA models in Multimedia Appendix 2. Finally, we used OLS multiple linear regression to verify any correlations with demographic and geographic statistics such as population, population density, and latitude [21].

### Concerning Multicomparison Adjustment Problem

The *P* value adjustment for a multicomparison test originates from the possibility of unintentionally increasing the number of false positives [22]. However, as shown by Greenland [23], the indiscriminate and unthinking implementation of this method can lead to conclusions that are erroneous, misleading, and, when sensitive topics are touched (eg, public health), even dangerous. Indeed, a scientist is called upon to consider both the consequences and the likelihood of incurring false results [23,24]. For instance, some authors suggest it is advisable to adjust the *P* values in exploratory investigations since the chances of spurious correlations due to the look-elsewhere effect are high [24]. Conversely, adjusting *P* values can be counterproductive when hypotheses are well-targeted and false negatives carry a serious risk (eg, airport metal detector). Nonetheless, Bender and Lange [25] highlighted that it is challenging to perform a multiple test adjustment in exploratory analyses due to the possible lack of a clear structure in the multiple tests; ergo, they recommend this procedure only for well-targeted hypotheses. Such a scenario spotlights the absence of a clear consensus [26]. Additional critical issues lie in the fact that the *P* value is not the probability that the test hypothesis is true nor that chance alone produced the observed association [27]. Ergo, adopting an (un)adjusted dichotomous threshold is not suitable for assessing the statistical significance of an outcome, as *P* values should be used—at best—as graded measures of the strength of evidence against the test hypothesis [27,28]. Finally, other authors have raised further concerns about adjusted *P* values. For example, Brandt [29] pointed out the medical unreasonableness of evaluating a patient's test results based on how many tests the patient had that day. With this provocation, Brandt [29] also questioned the scientific community about the possibility of dividing the results into different studies to bypass the problem of multicomparison. In conclusion, Greenland [23] stressed that proposing a single null hypothesis represents a bias in the analysis, and *P* values test not only the degree of data compatibility with the null hypothesis but all the test’s assumptions [27]. Hence, it must be admitted that every statistical interpretation or adjustment is strongly influenced by the authors’ prejudices and uncertainties on the assumptions made [23,24,27]. This is also true of the so-called “robust analyses,” whose complexity is further confusing. For these reasons, a scientist cannot do anything else beyond showing how methods and results vary under different conditions [23].

### Our Approach

This manuscript aimed to test statistical methods to identify epidemiologically relevant anomalies in a time series and provide near-definitive evidence on COVID-19 impact on mortality in Italy. Based on the evidence summarized in the previous subsection, we concluded that the best option was to give the reader the means to conduct an independent evaluation showing how results changed under different assumptions. Specifically, we used 2 approaches: The first, proposed by Siegel [30], involves the evaluation of the significance of a global test (ie, national population by sex) and then the implementation of other subtests (ie, regional population by sex) without corrections. In particular, we believe this approach is the most suitable for the purpose of this manuscript and denote it with A1. The second approach, denoted with A2, is the more conservative Holm-Bonferroni method with number of hypotheses m=47 [31].

## Results

### Overall Death Excess During 2020

**Figure 1:**
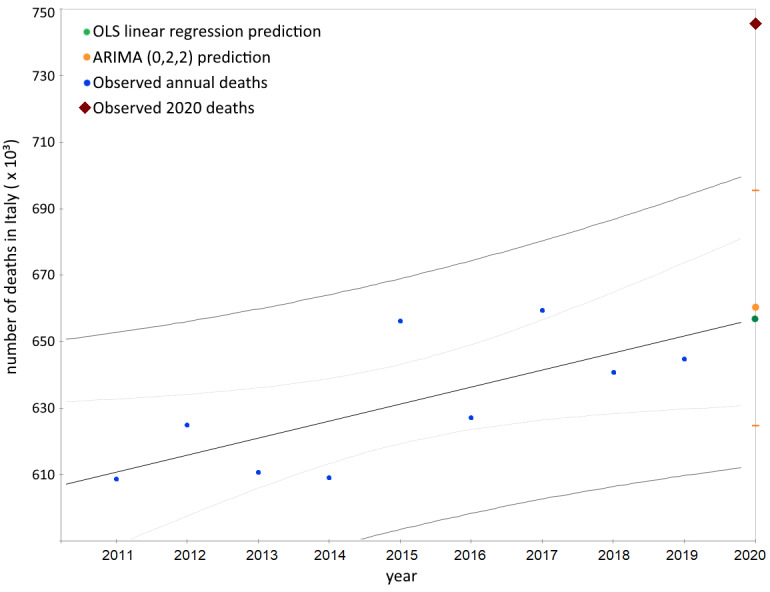
Annual number of deaths in Italy from 2011 to 2020: comparison between the observed value and the 2020 predictions of the ordinary least squares (OLS) linear regression and autoregressive integrated moving average (ARIMA; 0,2,2) models. The narrow bands represent the linear regression 95% CI of the mean value, while the wide bands represent the 95% CI of the observed values from 2011 to 2019. The orange dashes represent the 95% CI of the ARIMA prediction.

Compared with the OLS linear regression model prediction (Figure 1), the 2020 excess in the observed number of deaths in Italy was substantially larger (excess=89,287; % excess=13.6 [SE 5.3]). The detailed report is presented in Tables S1 and S2 in Multimedia Appendix 1. We found strong evidence supporting that the death increase in all regions was not due to chance (mean % excess=12.2 [SD 1.7]; t_21_=7.2; adjusted *P*<.001). Figure 1 also shows the high statistical confidence between the values predicted by the OLS linear regression and ARIMA (0,2,2) model; this constitutes further proof of the goodness of the linear interpolation.

### Male Mortality Rate During 2020

For A1, when the male mortality rate is considered, the 2020 excesses were large and highly significant in 13 of 21 regions (all *P*<.005). Moderate significant increases were observed in the other five regions (.02≤*P*≤.10). A low significance was obtained only in Molise, Basilicata, and Calabria (all *P*≥.20). Overall, the excess male mortality in Italy during 2020 was high and markedly significant (*P*<.001; excess=18.8 per 10,000; % excess=18.4 [SE 5.4]). Moreover, all regions recorded an excess male mortality between 5% (Basilicata) and 38% (Lombardy). Details of each region are provided in Table 1. Further information on the model goodness is provided in Table S3 in Multimedia Appendix 1. For A2, adjusted *P*≤.006 was reached nationally and in 6 regions (Piedmont, Lombardy, Trento, Veneto, Liguria, Emilia Romagna), while .02≤adjusted *P*≤.08 were reached in 5 regions (Bolzano, Friuli Venezia Giulia, Marche, Abruzzo, Apulia). Campania and Sardinia also registered a moderate significance (adjusted *P*≤.16). Adjusted *P*≥.43 were obtained in the remaining regions. Details of each region are provided in Table 1.

**Table 1:** Regional male mortality statistics: comparison of ordinary least squares (OLS) linear regression predicted mortality (predicted value) and observed mortality of 2020 (observed value) in Italy. The data are normalized to 10,000 people (deaths per 10,000).

Italian region	Predicted value	Predicted value SE	Observed value	Excess, % (SE)	*P* value^a^	Adjusted *P* value^a^
Italy	102.1	4.6	120.9	18.4 (5.4)	<.001	.006
Piemonte	105.7	5.2	132.3	25.1 (6.2)	<.001	<.001
Valle d’Aosta	114.4	11.3	136.3	19.1 (12.3)	.02	.40
Lombardia	98.5	4.1	136.2	38.3 (5.8)	<.001	<.001
Bolzano	93.6	4.9	110	17.5 (6.2)	.001	.02
Trento	90.7	4.7	121	33.4 (7)	<.001	<.001
Veneto	97.2	3.7	114.7	18 (4.5)	<.001	.002
Friuli Venezia Giulia	99.6	5.9	116.3	16.8 (7)	.002	.06
Liguria	103.4	5.6	126.5	22.3 (6.7)	<.001	.006
Emilia-Romagna	97.5	4.5	116.1	19.1 (5.6)	<.001	.006
Toscana	97.2	6	108.5	11.6 (7)	.03	.43
Umbria	94.5	6.3	105.4	11.5 (7.6)	.04	.62
Marche	96.3	5.4	111.1	15.3 (6.6)	.003	.07
Lazio	100.1	5.2	110.1	10 (5.7)	.02	.42
Abruzzo	101.9	4.5	114.6	12.5 (5)	.002	.06
Molise	107.3	7.2	113.8	6 (7.2)	.50	.99
Campania	116.1	5.5	129.9	11.9 (5.4)	.005	.12
Puglia	100.1	5.8	115.6	15.5 (6.7)	.003	.08
Basilicata	107.2	6.1	112.9	5.3 (6)	.40	.99
Calabria	106.5	6.1	113.9	6.9 (6.2)	.20	.99
Sicilia	112.7	7.2	122.9	9.1 (7)	.10	.99
Sardegna	101.2	5.3	113.7	12.3 (5.9)	.007	.16

^a^Grubbs test.

### Female Mortality Rate During 2020

For A1, highly significant excess female mortality was found in the northern regions and Sardinia (*P*≤.01, except Valle d’Aosta, *P*=.02). Moderately significant excesses were recorded in Tuscany, Marche, Molise, and Apulia (.04≤*P*≤.07). Scarcely significant differences were recorded in the rest of Italy (all *P*>.40). Nevertheless, all regions experienced an excess female mortality between 4% (Basilicata) and 31% (Lombardy). Details of each region are provided in Table 2. Further information on the model goodness is provided in Table S4 in Multimedia Appendix 1. For A2, Lombardy (adjusted *P*=.006) and Trento (adjusted *P*=.001) reached the greatest statistical significance. Moderate significance (.01*≤*adjusted *P*≤.06) was reached in 5 regions (Piedmont, Bolzano, Friuli Venezia Giulia, Liguria, and Emilia Romagna). Sardinia (adjusted *P*=.19) and Veneto (adjusted *P*=.20) also registered a modest significance. Low significance was observed in the remaining regions (all adjusted *P*≥.38)*.* Details of each region are provided in Table 2.

**Table 2:** Regional female mortality statistics: comparison of ordinary least squares (OLS) linear regression predicted mortality (predicted value) and observed mortality of 2020 (observed value) in Italy. The data are normalized to 10,000 people (deaths per 10,000).

Italian region	Predicted value	Predicted value SE	Observed value	Excess, % (SE)	*P* value^a^	Adjusted *P* value^a^
Italy	68.3	3.9	77.9	14.1 (6.6)	.005	.12
Piemonte	70.8	4	84.1	18.8 (6.8)	.001	.02
Valle d’Aosta	69.9	9.8	88.9	27.1 (19.3)	.02	.38
Lombardia	64.3	3.5	84.2	30.9 (7.2)	<.001	.006
Bolzano	60.5	3.6	73.9	22.1 (7.4)	<.001	.01
Trento	59.4	2.8	73.4	23.6 (5.9)	<.001	.001
Veneto	64.2	3.8	72.8	13.4 (6.9)	.009	.20
Friuli Venezia Giulia	64.2	2.8	72.6	13 (5)	.001	.05
Liguria	67.4	4.3	79.3	17.7 (7.6)	.002	.06
Emilia-Romagna	66.1	3.3	75.6	14.4 (5.7)	.002	.05
Toscana	65.4	3.7	71.2	8.9 (6.3)	.07	.80
Umbria	63.2	4	67	6.1 (6.8)	.43	.99
Marche	64	4.7	71.8	12.2 (8.5)	.05	.68
Lazio	67.8	4.7	71.9	6 (7.5)	.56	.99
Abruzzo	67.6	4.6	72	6.5 (7.4)	.43	.99
Molise	66.4	5.1	74.7	12.6 (8.9)	.06	.73
Campania	80.3	5.7	85.1	6 (7.7)	N/A^b^	N/A
Puglia	68.5	4.7	76.5	11.6 (7.8)	.04	.65
Basilicata	71.5	4.7	74.4	4.1 (7)	.40	.99
Calabria	71.7	4.4	75.1	4.8 (6.5)	.79	.99
Sicilia	78	5.9	83.4	7 (8.3)	.47	.99
Sardegna	64	3.4	71.5	11.6 (5.9)	.008	.19

^a^Grubbs test.

^b^N/A: not available.

### Relationship Between Deaths and Geographical-Demographic Statistics

The linear multiregression model among the log-transformed statistics, the regional number of inhabitants (X_1_), regional population density (X_2_), regional latitude (X_3_), and 2020 regional excess deaths (Y), returned the following equation:

Y=f(X_3_)=k×pow(X_3_, a),

with k=2.6×10^-7^, a=9.9, *R*=0.82, adjusted *P*<.001.

### Retrospective Analysis of Deaths

**Figure 2:**
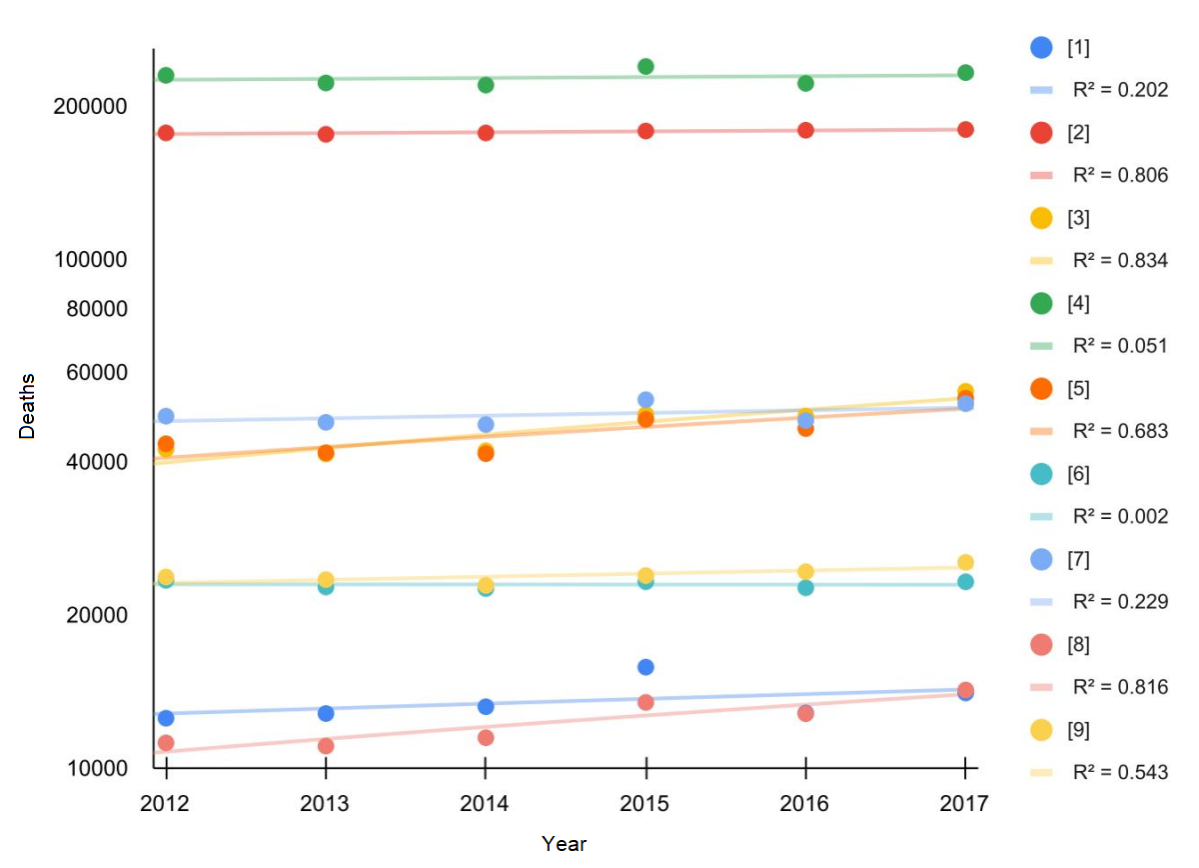
Number of deaths per cause of death from 2011 to 2017 in Italy; the most updated National Institute of Statistics (ISTAT) data were available until 2017 (see Multimedia Appendix 1). 1: infectious and parasitic diseases; 2: tumors; 3: psychic disorders, diseases of the nervous system and organs of the senses; 4: diseases of the circulatory system; 5: diseases of the respiratory system; 6: diseases of the digestive system; 7: other morbid states; 8: poorly defined symptoms, signs, and morbid states; 9: external causes of trauma and poisoning.

Figure 2 shows the number of deaths per cause of death from 2012 to 2017 in Italy (2018 and 2019 data were not available, as shown in Multimedia Appendix 1). Tumors and diseases of the circulatory system always accounted for over 60% of total deaths (also considering the projections for 2020). The percentages of male (female) deaths for tumors ranged from 55.6% (44.4%) to 56.3% (43.7%), while deaths related to the circulatory system were 43.1% (56.9%) to 43.7% (56.3%). All trends were markedly linear.

**Figure 3:**
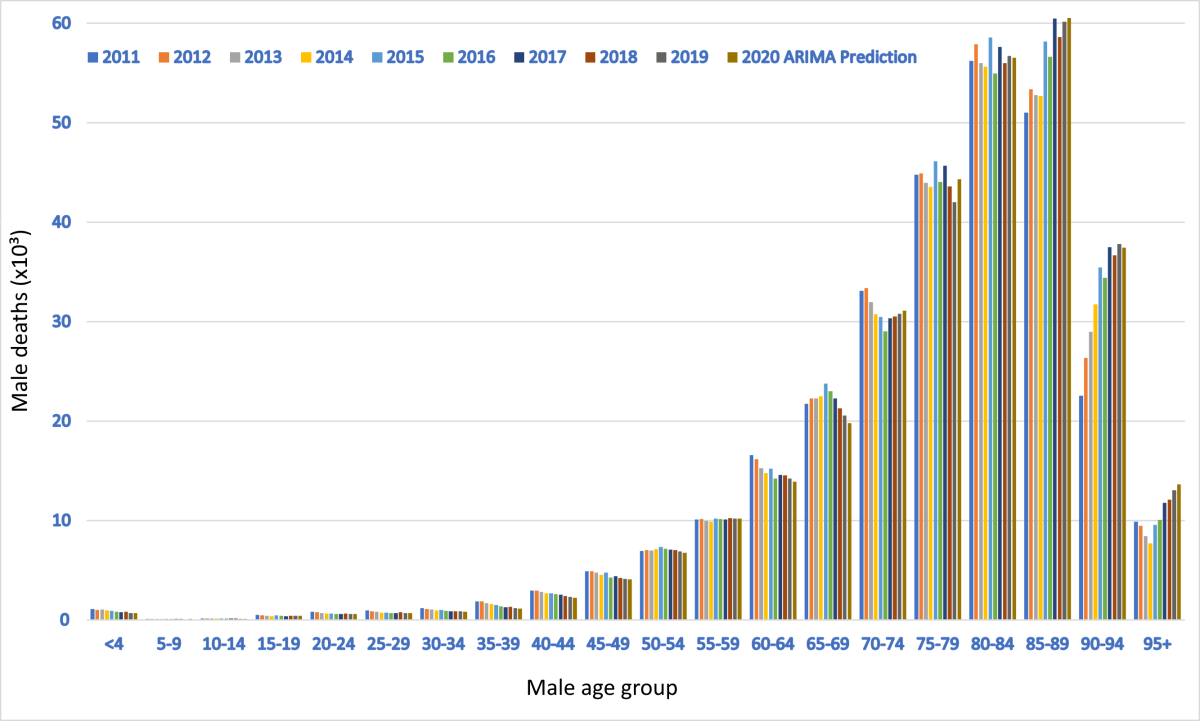
Male deaths per age group in Italy from 2011 to 2019 and autoregressive integrated moving average (ARIMA) predictions for 2020.

**Figure 4:**
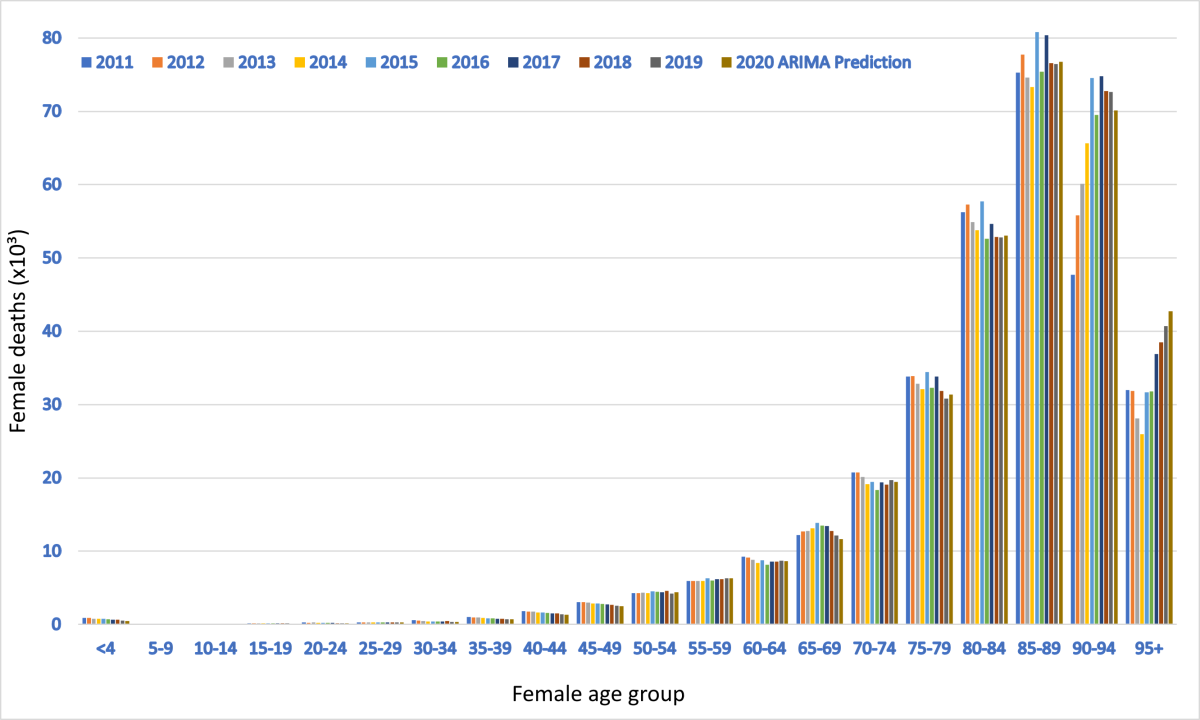
Female deaths per age group in Italy from 2011 to 2019 and autoregressive integrated moving average (ARIMA) predictions for 2020.

Finally, Figure 3 and Figure 4 show male and female deaths, respectively, by age group from 2011 to 2019. Explicitly calculating each trend for each age and sex group and summing the predictions for 2020, we obtained the best value of 648,733 deaths. All trends were markedly linear (Figures S3 and S4 in Multimedia Appendix 1). Summing up all the forecasts of the ARIMA models for each age and sex group, we obtained a total of 637,534 deaths. A similar result was obtained by summing the global trends for men and women (640,508 deaths).

### Comparison Between Male and Female Mortality

The increases in mortality were 18% (*P*<.001; adjusted *P*=.006) for men and 14% (*P*=.005; adjusted *P*=.12) for women at the national level and 16% for men compared with 13% for women, on average, at the regional level. However, we found limited significance when comparing the residuals' populations using the Mann-Whitney *U* test (*P*=.27; adjusted *P*=.99).

## Discussion

### Principal Findings

This paper provides strong evidence in favor of an anomalous mortality event during 2020 in Italy, which was not predictable based on endogenous causes such as deaths and mortality trends between 2011 and 2019. Notably, the number of total deaths observed in 2020 exceeded the linear regression model prediction by more than 89,000 (a value nearly 3 times greater than the prediction standard error) and the ARIMA prediction by more than 86,000. Grubbs and *t* tests confirmed that this figure was unexpected. At the national level, the increase in mortality was 18% for the male population and 14% for the female population. Nonetheless, the statistical significance of this difference was low. The total excess mortality was positively correlated with latitude, which explained the data set variability much better than demographic statistics like population number and density. All the “deceases due to causes of death” trends from 2012 to 2017 were appreciably linear or stationary; this precludes the existence of anomalous subtrends linked to the causes of death. Moreover, summing up all the 2020 death predictions by age group, we obtained a value ranging from 640,000 to 660,000 deceased, significantly far from the observed one (750,000). In conclusion, these findings confirm the absence of any confounding inner subtrends capable of explaining the excess deaths during 2020 in Italy.

### Comparison With Prior Work

To the best of our knowledge, the most comprehensive and detailed study examining excess mortality during 2020 in Italy was the report redacted by the ISTAT and National Institute of Health (ISS) [32]. Their research focuses on comparing the March-December 2015-2019 and 2020 periods, starting from the assumption that COVID-19 is the cause of the discrepancies observed. On the contrary, our analysis has been more impartial since we have not introduced any hypothesis about the reasons that caused this phenomenon. Therefore, our findings provide evidence of statistical and epidemiological significance that had not been considered before. Specifically, excluding internal causes gives further strength to the theories that identify COVID-19 as the principal cause of such a tragic scenario. COVID-19 dangerousness is confirmed at the molecular-genetic level [33-36]. The strong positive correlation we found between excess mortality and latitude is compatible with greater virulence and mortality of COVID-19 in northern Italy depicted by other literature [37,38]. In this regard, an increasing number of mathematical-statistical investigations classify COVID-19 as a seasonal low-temperature infection [39-41], although the effect size of the environmental factors is still debated [42]. However, it is a fact that low temperatures can have indirect effects on the spread of infections, like the creation of indoor gatherings—with insufficient air circulation—and the weakening of the immune defenses [43,44]. Since average temperatures in northern regions are lower than the rest of the peninsula [45], this phenomenon could partially explain the Italian epidemiological scenario. A large amount of literature has also identified pollution as a relevant COVID-19 risk factor. For instance, NO_2_, PM10, and PM2.5 were causally connected with more serious situations, as they can drastically reduce the immune response and compromise respiratory functions [45-48]. This type of pollutant is widespread in the Po Valley [45,46]. Contrary to other literature, our paper did not detect a high significance in the difference between male and female national mortalities [49-58]. Nonetheless, this result is not conclusive and deserves further investigation as such a discrepancy could be more evident by considering the most affected and exposed age groups. Moreover, the COVID-19 course is influenced by numerous comorbidities, such as cancer, chronic kidney diseases, diabetes mellitus, hypertension, chronic obstructive pulmonary diseases, asthma, chronic respiratory diseases, immunocompromised state, HIV infection, heart conditions, overweight and obesity, dementia or other neurological conditions, and mental health conditions [59-61]. The majority of these pathologies are more common in older age groups, which helps explain the greater aggressiveness of the infection in some regions [17,48]. Hence, it is necessary to consider that the prepandemic epidemiological scenario has contributed to enhancing the disease damage in Italy. Nevertheless, it would be incorrect to consider only the older population as vulnerable: Phenomena such as long COVID (ie, the onset of medical complications that last weeks to months after initial recovery) are increasing in younger age groups, including children and adolescents [62,63]. The most common symptoms of long COVID are fatigue, weakness, cough, chest tightness, breathlessness, palpitations, myalgia, and difficulty focusing; their appearance is not related to the severity of the COVID-19 course [63,64]. Moreover, new variants of concern—favored by the uncontrolled spread of the virus—continuously pose new threats to all age groups [33,65,66]. In this regard, strategies such as vaccinations and nonpharmaceutical containment measures have been and continue to be fundamental to control COVID-19 diffusion, avoid hospital overcrowding, and slow down the epidemiological peaks [67-73]. Indeed, although this paper has provided evidence in favor of a high number of deaths due to COVID-19 in Italy during 2020 (before the administration of COVID-19 vaccines), lockdowns, social distancing, and masks have prevented the death toll from being numerous times higher [74-77].

### Limitations

Our approach has limitations to be considered. Since statistical significance is a measure of data compatibility with the null hypothesis (including the model’s assumptions), the evidence provided in this paper could vary under different initial hypotheses. However, the degree of uncertainty was reduced by targeting the tested hypotheses well. Furthermore, causal relationships have not been directly investigated. Therefore, these findings must be contextualized in light of the results of other literature. Finally, the discrepancies between the model predictions and the observed data were not weighted on the clinical characteristics of the patients.

### Conclusions

This paper provides strong evidence on the absence of historical endogenous reasons capable of explaining the anomalous mortality increase recorded in Italy during 2020. Weighing these statistical results on the numerous molecular-genetic, medical, biological, virological, and epidemiological-based publications that confirmed high COVID-19 virulence, we conclude that the pandemic impact on excess deaths in Italy constitutes a scientific fact. This answers the question “Died from COVID or died with COVID?” Specifically, this manuscript can be adopted by health authorities and disclosure agencies to discredit fake news that minimizes the COVID-19 risk. Moreover, given the marked concordance between ARIMA and OLS regression models, we suggest that these methods be exploited for public health surveillance aims. In particular, considering their efficiency and effectiveness, it is possible to derive meaningful information regarding current and future epidemiological situations from the comparison between the predicted and observed trends.

## Appendix

Multimedia Appendix 1 COVID-19 Mortality Italy, Rovetta.

Multimedia Appendix 2 Autoregressive integrated moving average (ARIMA) models.

## Abbreviations

ARIMA: autoregressive integrated moving average
ISS: National Institute of Health
ISTAT: National Institute of Statistics
OLS: ordinary least squares
SARIMA: ARIMA + seasonal component
SIR: Susceptible-Infected-Recovered
