# Supplementary material for: The Impact of COVID-19 on Mortality in Italy: Retrospective Analysis of Epidemiological Trends
Source: JMIR Public Health Surveill. 2022 Apr 7;8(4):e36022. doi: 10.2196/36022 (PMC8993143; doi:10.2196/36022)
Supplement: Multimedia Appendix 1 [file publichealth_v8i4e36022_app1.docx]

**1. Data collection details**

[Number of deaths by age group]. Each age group has been manually selected from the website: [http://dati.istat.it/Index.aspx?DataSetCode=DCIS_MORTALITA1#](http://dati.istat.it/Index.aspx?DataSetCode=DCIS_MORTALITA1) (item: mortalità - decessi - morti). The datasets have been divided into male, female, and total. Investigated period: 2011-2019. We have left all the remaining parameters to the default ones.

[Number of deaths]. We used the following Excel file already paginated by the national health observatory: <https://www.osservatoriosullasalute.it/wp-content/uploads/2021/05/ro-2020-isc-covid.xlsx>. The datasets have been divided into male, female, and total. Investigated period: 2011-2020.

[Number of deaths by death causes]. The data were manually extracted from item "Morti per classe di età, sesso e gruppo di cause" of the following reports of the National Statistical Institute (ISTAT): <https://www.istat.it/it/files//2020/12/C04.pdf>, <https://www.istat.it/it/files//2019/12/C04.pdf>, <https://www.istat.it/it/files//2018/12/C04.pdf>, <https://www.istat.it/it/files//2017/12/C04.pdf>, <https://www.istat.it/it/files//2016/12/C04.pdf>, <https://www.istat.it/it/files//2015/12/C04.pdf>. The datasets have been divided into male, female, and total. Investigated period: 2012-2017.

[Population number per age group]. The data were manually extracted from: <https://www.tuttitalia.it/statistiche/popolazione-eta-sesso-stato-civile-2019/>. The datasets have been divided into male, female, and total. Investigated period: 2011-2019.

[Population number and density per region]. The data were manually extracted from: <https://www.tuttitalia.it/regioni/densita/>. Investigated period: 2020.

All websites were consulted on November 01, 2021.

**2. Linear trends**

f(x) = ax+b, g(x) = cx+d | f(x)+g(x) = ax+b+cx+d = (a+c)x+(b+d) = ex+f.


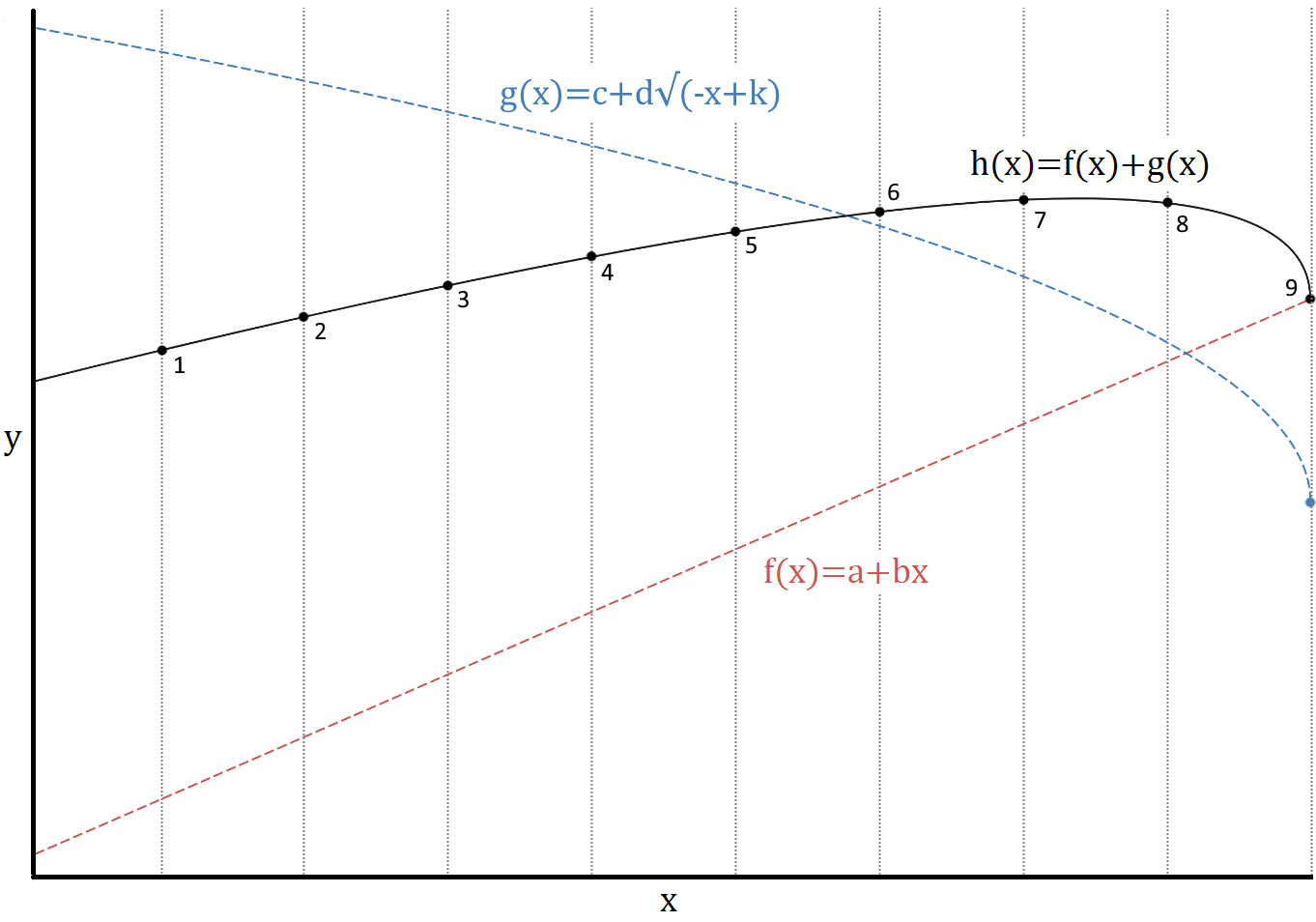


**Figure S1.** Example of a statistical anomaly due to the superposition of an increasing linear trend and a decreasing sublinear trend. Indeed, measure number 9 is very far from the prediction of a model built or trained on the first eight values. However, such an anomaly is not due to any external factor (ie, the reason is endogenous). Such a scenario could be even more complex to decipher if there are multiple trends to be considered.

**3. Procedure and statistical analysis details**

We modeled regional trends in annual deaths and mortality from 2011 to 2019 through ordinary least squares (OLS) linear regression. The standard assumptions for this regression model were automatically checked by the *XLSTAT Linear Regression* tool (default settings). However, a qualitative graphic check was also performed to observe any non-normality of the residuals, heteroskedasticity, or outliers. Calling *Δ** the residuals' dataset from 2011 to 2019 and *Δ* the residuals' dataset from 2011 to 2020, we performed the following steps: i) we evaluated the distribution normality of *Δ** through the Shapiro-Wilk test plus Q-Q and Box plots, ii) we evaluated the distribution normality of *Δ* through the Shapiro-Wilk test plus Q-Q and Box plots, iii) through the Grubbs one sided test plus a graphical check, we searched for high outliers in *Δ**, and iv) through the Grubbs one sided test plus a graphical check, we searched for outliers in *Δ*. In this way, we assessed whether the residual *δ_2020* belonged to the *Δ** distribution (ie, if there was an anomaly in national and regional deaths during 2020 compared to 2011-2019). The Grubbs test was performed operating the *RStudio v.4.1.2* software (library *outliers*). We also performed a one-sample t-test to assess if the regional death increases were due to chance. This was done by comparing the 2020 excess deaths population to a fixed null mean (i.e., the expected residual). The hypothesis of normality was verified with the same methods as above. Furthermore, we calculated the difference between the model prediction and the observed value. Standard errors or 95% confidence intervals were reported alongside each measurement to allow the reader for independent data review. To validate or deny any statistical anomalies in the number of deaths during 2020, we checked all the trends of the following annual statistics within the 2011-2019 time frame: male deaths by age group, female deaths by age group, male mortality by age group, female mortality by age group, deaths by causes of death, male deaths by causes of death, female deaths by causes of death. Specifically, we searched for anomalous non-linear sub-trends capable of distorting the interpretations on the cumulative data (indeed, sum of linear trends is linear as shown above). An example of this phenomenon is shown in Figure S1. Concerning male and female deaths for age groups, we also calculated the 2020 forecast for each age group through an *ARIMA (p, d, q)* model using the *RStudio v.4.1.2* software (libraries *forecast* and *tseries*). The *d* parameter has been set equal to the number of differentiations necessary to sufficiently stationary the series. The series stationarity was evaluated through the Augmented Dickey-Fuller test, the Mann-Kendall test (to highlight possible trends), and a graphical control. The *p* and *q* parameters were chosen by examining autocorrelation graphs (ACF) and partial autocorrelation (PACF) graphs and then minimizing the Akaike Information Criteria (AIC) value. To facilitate the reproducibility of the analysis, we have provided all the ARIMA models in Supplementary File 2. Finally, we used OLS multiple linear regression to verify any correlations with demographic and geographic statistics such as population, population density, and latitude. The standard assumptions of the model (i.e., normality of the residuals, homoskedasticity, absence of outliers, and absence of multicollinearity) were automatically verified by the *Multiple Linear Regression Calculator* tool by *Statistics Kingdom*.

**4. Supplementary Figures and Tables.**

|  | PRE | S.E. PRE | OBS | % EXC | S.E. % EXC |
| --- | --- | --- | --- | --- | --- |
| Italy | 656859 | 30563 | 746146 | 13.6 | 5.3 |
| Piemonte | 55070 | 2600 | 66054 | 19.9 | 5.7 |
| Valle d’Aosta | 1523 | 135 | 1849 | 21.4 | 11.1 |
| Lombardia | 102865 | 4165 | 136249 | 32.5 | 5.4 |
| Bolzano | 4590 | 175 | 5458 | 18.9 | 4.6 |
| Trento | 5246 | 167 | 6626 | 26.3 | 4 |
| Veneto | 50680 | 2077 | 57836 | 14.1 | 4.7 |
| Friuli Venezia Giulia | 14665 | 659 | 16617 | 13.3 | 5.1 |
| Liguria | 22023 | 1189 | 25827 | 17.3 | 6.4 |
| Emilia-Romagna | 51508 | 2309 | 59665 | 15.8 | 5.2 |
| Toscana | 44603 | 2445 | 48135 | 7.9 | 6 |
| Umbria | 10545 | 654 | 11131 | 5.6 | 6.6 |
| Marche | 18061 | 1159 | 20123 | 11.4 | 7.3 |
| Lazio | 60036 | 2952 | 62161 | 3.5 | 5.1 |
| Abruzzo | 15375 | 822 | 16296 | 6 | 5.7 |
| Molise | 3865 | 264 | 4127 | 6.8 | 7.4 |
| Campania | 56164 | 3122 | 59425 | 5.8 | 6 |
| Puglia | 40770 | 2312 | 44650 | 9.5 | 6.3 |
| Basilicata | 6654 | 284 | 6839 | 2.8 | 4.4 |
| Calabria | 20837 | 1125 | 21331 | 2.4 | 5.6 |
| Sicilia | 54351 | 3238 | 56753 | 4.4 | 6.3 |
| Sardegna | 17427 | 676 | 18994 | 9 | 4.3 |

**Supplementary Table 1.** Comparison between prediction and the actual number of deaths during 2020 in all Italian regions. Legend: PRE = predicted value, S.E. = standard error, OBS = observed value, % EXC = percentage excess

|  | F P | R² | S.E. R² | Δ* SW P | Δ SW P |
| --- | --- | --- | --- | --- | --- |
| Italy | 0.034 | 0.495 | 0.19 | 0.274 | 0.005 |
| Piemonte | 0.012 | 0.615 | 0.161 | 0.867 | <.001 |
| Valle d’Aosta | 0.043 | 0.464 | 0.195 | 0.178 | 0.009 |
| Lombardia | 0.003 | 0.744 | 0.118 | 0.908 | <.001 |
| Bolzano | 0.002 | 0.765 | 0.152 | 0.165 | <.001 |
| Trento | 0.001 | 0.795 | 0.098 | 0.74 | <.001 |
| Veneto | 0.009 | 0.645 | 0.152 | 0.718 | 0.003 |
| Friuli Venezia Giulia | 0.933 | 0.001 | 0.177 | 0.778 | 0.009 |
| Liguria | 0.883 | 0.003 | 0.029 | 0.709 | 0.005 |
| Emilia-Romagna | 0.069 | 0.398 | 0.203 | 0.627 | 0.002 |
| Toscana | 0.228 | 0.2 | 0.191 | 0.325 | 0.192 |
| Umbria | 0.514 | 0.063 | 0.126 | 0.249 | 0.205 |
| Marche | 0.16 | 0.261 | 0.202 | 0.052 | 0.031 |
| Lazio | 0.035 | 0.494 | 0.19 | 0.02 | 0.016 |
| Abruzzo | 0.428 | 0.092 | 0.147 | 0.042 | 0.049 |
| Molise | 0.954 | 0.001 | 0.017 | 0.025 | 0.039 |
| Campania | 0.11 | 0.323 | 0.206 | 0.023 | 0.022 |
| Puglia | 0.029 | 0.519 | 0.185 | 0.268 | 0.129 |
| Basilicata | 0.008 | 0.658 | 0.148 | 0.616 | 0.699 |
| Calabria | 0.091 | 0.355 | 0.205 | 0.12 | 0.223 |
| Sicilia | 0.149 | 0.273 | 0.203 | 0.478 | 0.297 |
| Sardegna | 0.001 | 0.796 | 0.097 | 0.817 | 0.052 |

**Supplementary Table 2.** Goodness-of-fit statistics for total excess deaths during 2020. Legend: F P = F-statistic P-value, S.E. = standard error, SW P = Shapiro-WIlk P-value.

|  | F P | R² | S.E. R² | Δ* SW P | Δ SW P |
| --- | --- | --- | --- | --- | --- |
| Italy | <.001 | 0.866 | 0.067 | 0.799 | 0.001 |
| Piemonte | 0.001 | 0.798 | 0.096 | 0.819 | <.001 |
| Valle d’Aosta | 0.28 | 0.164 | 0.181 | 0.334 | 0.084 |
| Lombardia | <.001 | 0.897 | 0.052 | 0.717 | <.001 |
| Bolzano | <.001 | 0.844 | 0.077 | 0.077 | 0.001 |
| Trento | <.001 | 0.887 | 0.057 | 0.403 | <.001 |
| Veneto | <.001 | 0.924 | 0.039 | 0.636 | 0.003 |
| Friuli Venezia Giulia | <.001 | 0.888 | 0.056 | 0.379 | 0.003 |
| Liguria | 0.001 | 0.817 | 0.088 | 0.014 | <.001 |
| Emilia-Romagna | 0.001 | 0.824 | 0.085 | 0.295 | <.001 |
| Toscana | 0.002 | 0.776 | 0.105 | 0.954 | 0.22 |
| Umbria | 0.001 | 0.823 | 0.086 | 0.349 | 0.214 |
| Marche | 0.002 | 0.782 | 0.103 | 0.401 | 0.007 |
| Lazio | <.001 | 0.867 | 0.066 | 0.975 | 0.244 |
| Abruzzo | <.001 | 0.903 | 0.049 | 0.08 | 0.004 |
| Molise | 0.002 | 0.753 | 0.115 | 0.213 | 0.198 |
| Campania | <.001 | 0.85 | 0.074 | 0.313 | 0.005 |
| Puglia | 0.001 | 0.804 | 0.094 | 0.299 | 0.009 |
| Basilicata | 0.01 | 0.636 | 0.155 | 0.967 | 0.864 |
| Calabria | 0.005 | 0.7 | 0.134 | 0.248 | 0.39 |
| Sicilia | 0.007 | 0.674 | 0.143 | 0.5 | 0.298 |
| Sardegna | 0.001 | 0.822 | 0.086 | 0.726 | 0.058 |

**Supplementary Table 3.** Goodness-of-fit statistics for excess male mortality during 2020. Legend: F P = F-statistic P-value, S.E. = standard error, SW P = Shapiro-WIlk P-value.

|  | F P | R² | S.E. R² | Δ* SW P | Δ SW P |
| --- | --- | --- | --- | --- | --- |
| Italy | 0.008 | 0.659 | 0.148 | 0.813 | 0.027 |
| Piemonte | 0.041 | 0.47 | 0.194 | 0.757 | 0.003 |
| Valle d’Aosta | 0.326 | 0.137 | 0.171 | 0.061 | 0.015 |
| Lombardia | 0.003 | 0.733 | 0.201 | 0.799 | <.001 |
| Bolzano | 0.002 | 0.778 | 0.105 | 0.065 | <.001 |
| Trento | 0.004 | 0.717 | 0.128 | 0.5 | <.001 |
| Veneto | 0.017 | 0.579 | 0.066 | 0.867 | 0.06 |
| Friuli Venezia Giulia | <.001 | 0.87 | 0.065 | 0.643 | 0.01 |
| Liguria | 0.008 | 0.661 | 0.147 | 0.657 | 0.015 |
| Emilia-Romagna | 0.01 | 0.632 | 0.156 | 0.752 | 0.007 |
| Toscana | 0.009 | 0.644 | 0.153 | 0.844 | 0.575 |
| Umbria | 0.008 | 0.654 | 0.149 | 0.113 | 0.189 |
| Marche | 0.081 | 0.372 | 0.205 | 0.023 | 0.027 |
| Lazio | 0.004 | 0.708 | 0.131 | 0.644 | 0.85 |
| Abruzzo | 0.012 | 0.62 | 0.16 | 0.239 | 0.004 |
| Molise | 0.01 | 0.637 | 0.155 | 0.529 | 0.274 |
| Campania | 0.023 | 0.544 | 0.18 | 0.236 | 0.182 |
| Puglia | 0.009 | 0.643 | 0.153 | 0.63 | 0.273 |
| Basilicata | 0.188 | 0.233 | 0.198 | 0.474 | 0.626 |
| Calabria | 0.015 | 0.594 | 0.167 | 0.338 | 0.395 |
| Sicilia | 0.038 | 0.482 | 0.192 | 0.23 | 0.58 |
| Sardegna | 0.003 | 0.749 | 0.116 | 0.744 | 0.056 |

**Supplementary Table 4.** Goodness-of-fit statistics for excess female mortality during 2020. Legend: F P = F-statistic P-value, S.E. = standard error, SW P = Shapiro-WIlk P-value.


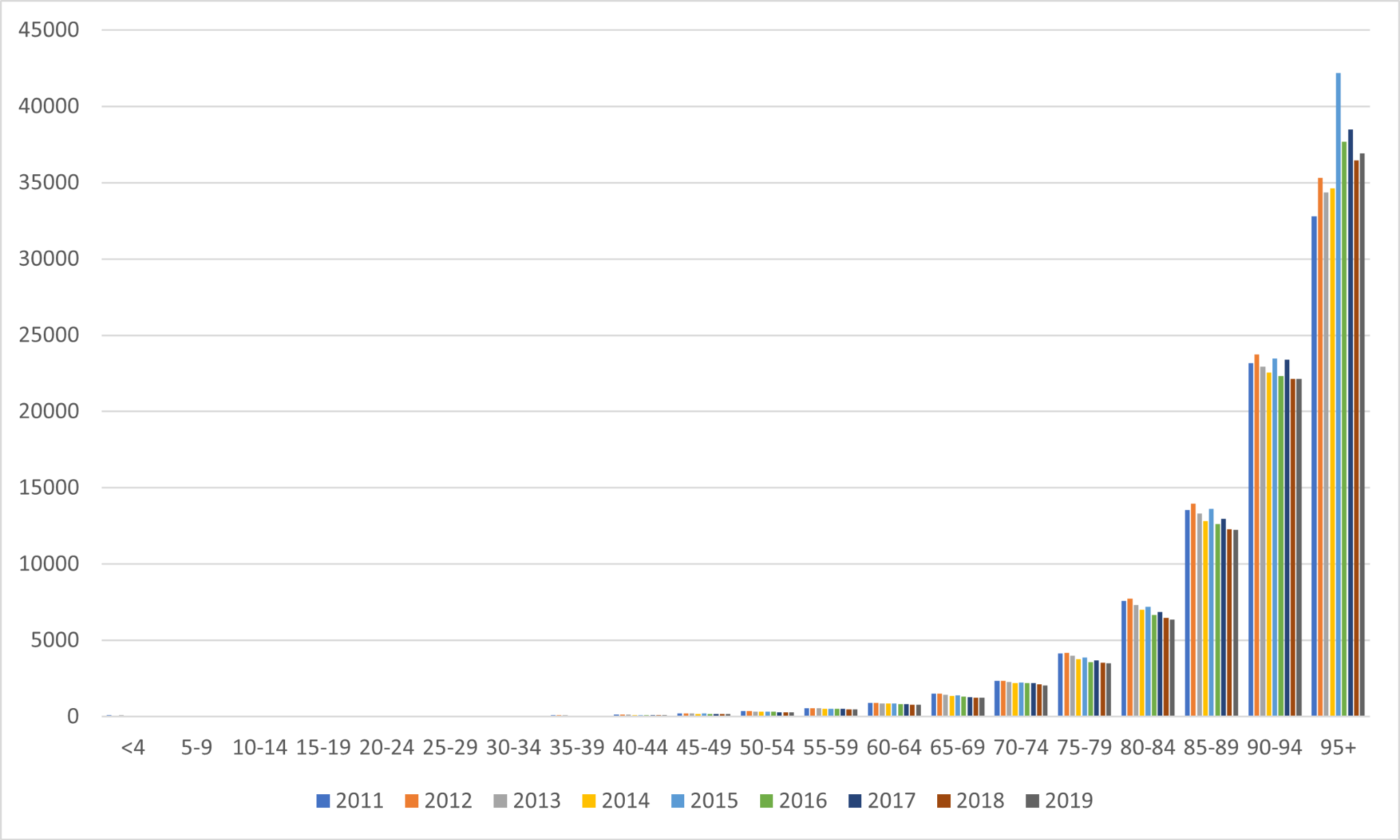


**Figure S2.** Male deaths per 100,000 inhabitants per age group from 2011 to 2019 (Italy).


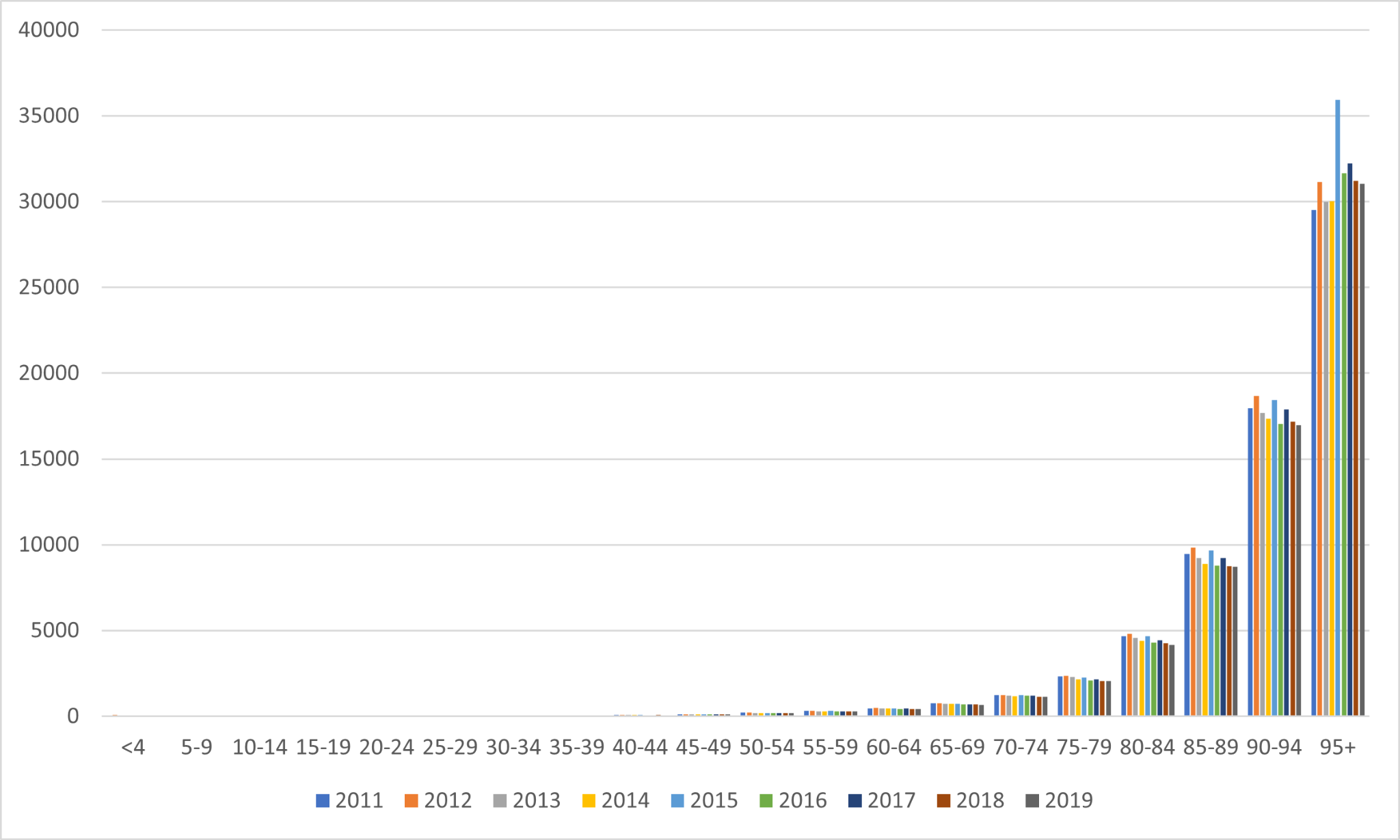


**Figure S3.** Female deaths per 100,000 inhabitants per age group from 2011 to 2019 (Italy).


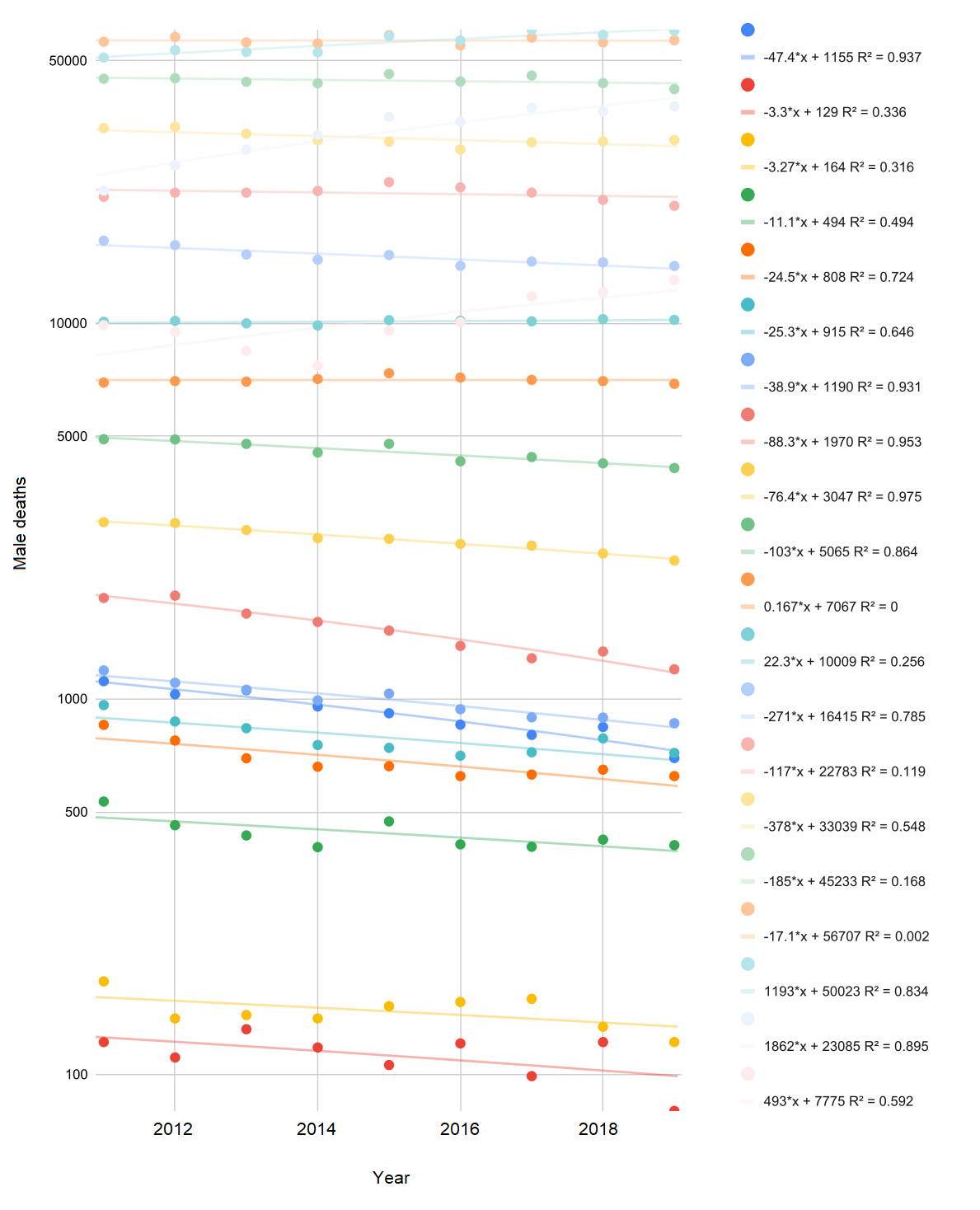


**Figure S4.** Male deaths per age group.

**
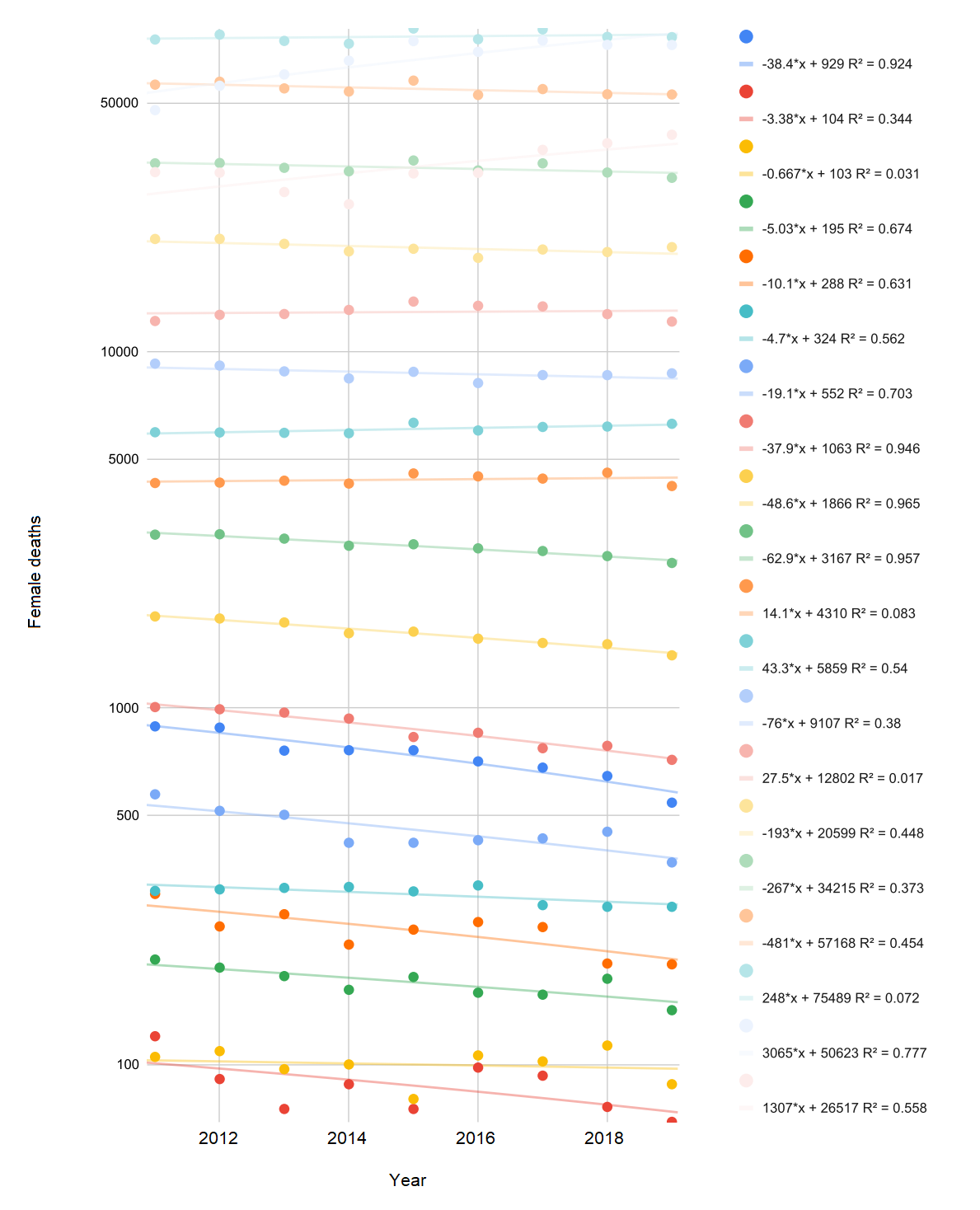
**

**Figure S5.** Female deaths per age group.
