# Supplementary figures and images for: The Impact of COVID-19 on Mortality in Italy: Retrospective Analysis of Epidemiological Trends
Source: JMIR Public Health Surveill. 2022 Apr 7;8(4):e36022. doi: 10.2196/36022 (PMC8993143; doi:10.2196/36022)

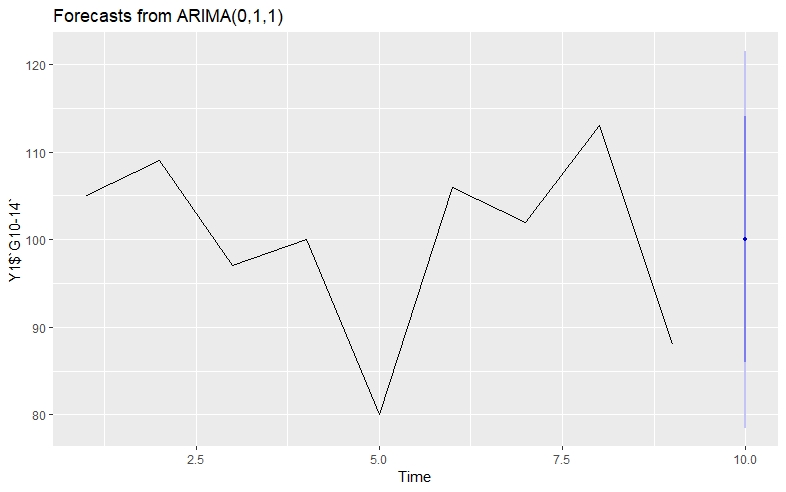

Supplement: Multimedia Appendix 2 [file publichealth_v8i4e36022_app2.zip › Female/F10-14.jpeg]

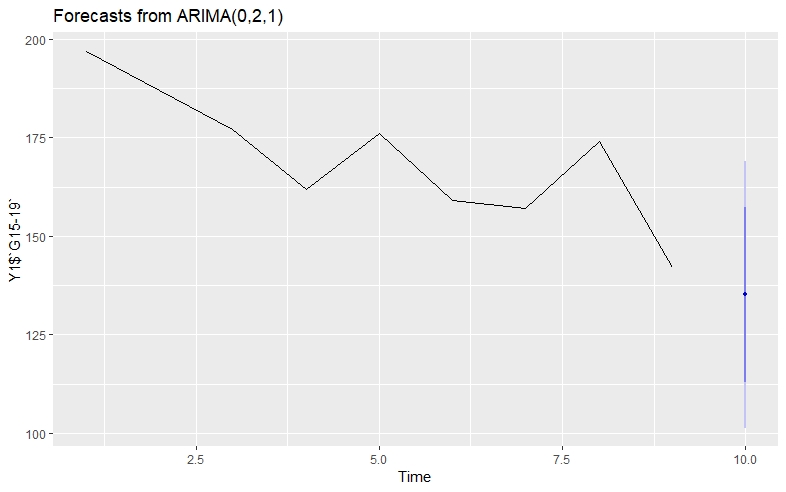

Supplement: Multimedia Appendix 2 [file publichealth_v8i4e36022_app2.zip › Female/F15-19.jpeg]

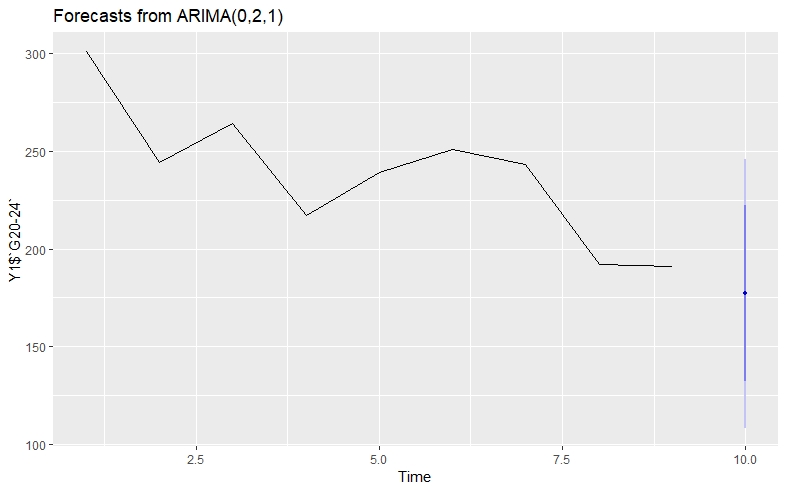

Supplement: Multimedia Appendix 2 [file publichealth_v8i4e36022_app2.zip › Female/F20-24.jpeg]

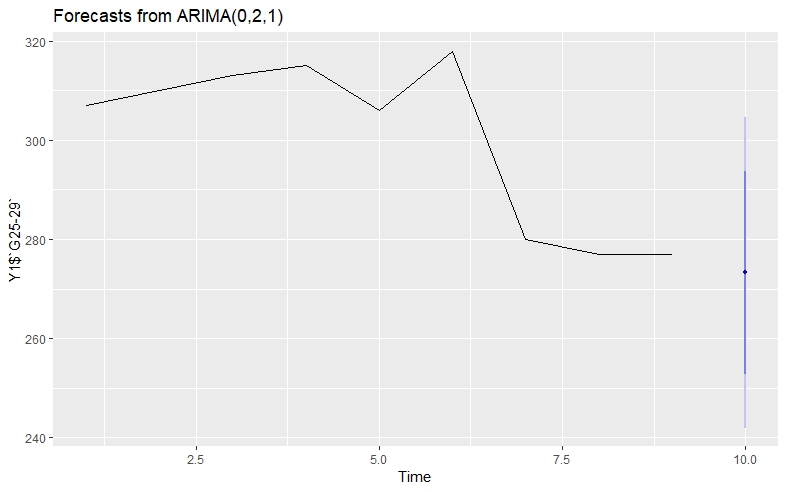

Supplement: Multimedia Appendix 2 [file publichealth_v8i4e36022_app2.zip › Female/F25-29.jpeg]

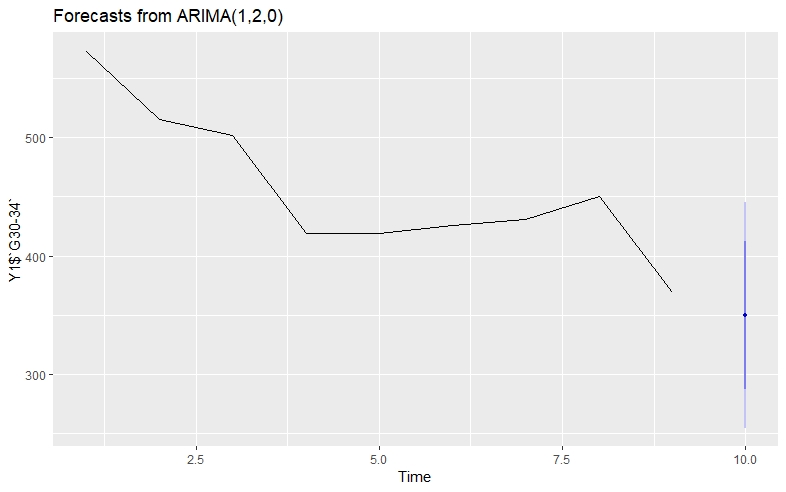

Supplement: Multimedia Appendix 2 [file publichealth_v8i4e36022_app2.zip › Female/F30-34.jpeg]

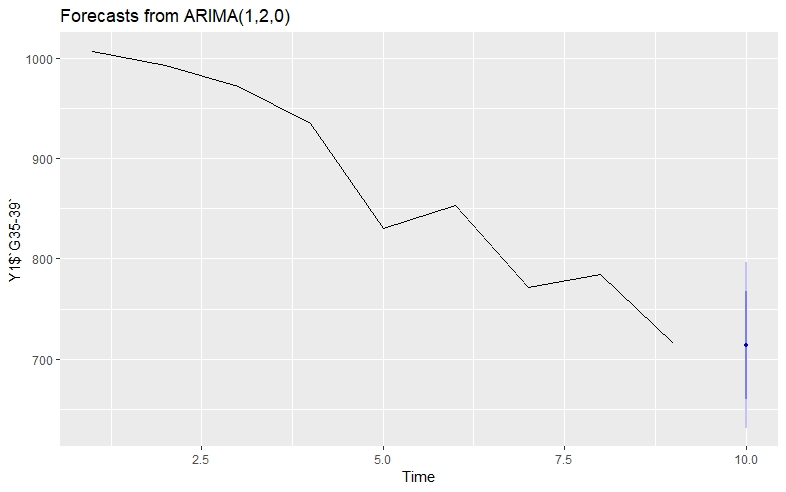

Supplement: Multimedia Appendix 2 [file publichealth_v8i4e36022_app2.zip › Female/F35-39.jpeg]

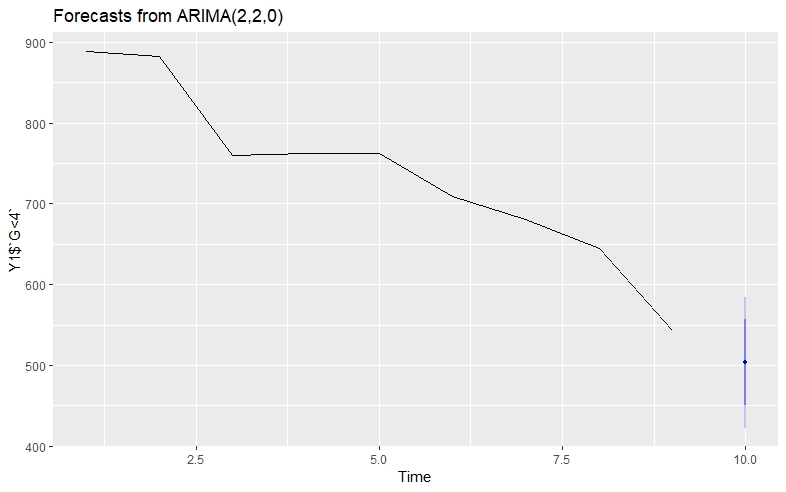

Supplement: Multimedia Appendix 2 [file publichealth_v8i4e36022_app2.zip › Female/F4-.jpeg]

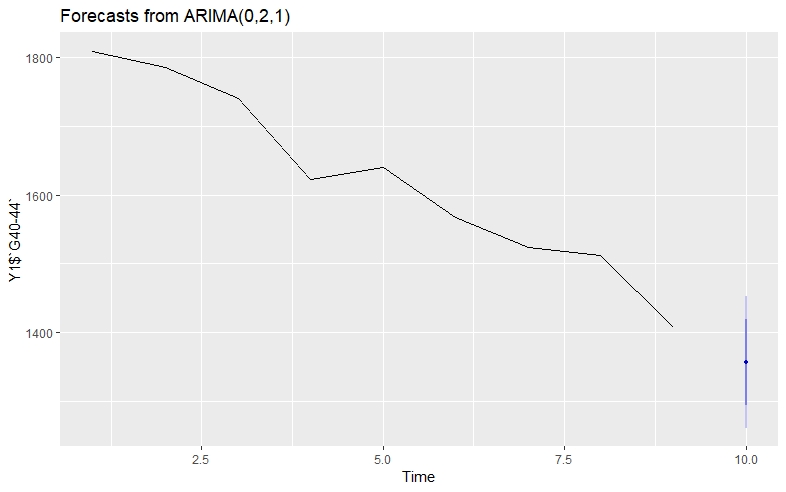

Supplement: Multimedia Appendix 2 [file publichealth_v8i4e36022_app2.zip › Female/F40-44.jpeg]

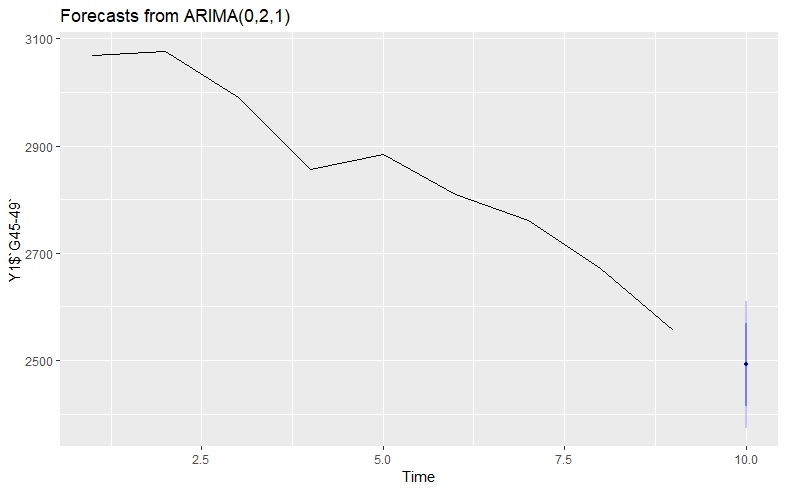

Supplement: Multimedia Appendix 2 [file publichealth_v8i4e36022_app2.zip › Female/F45-49.jpeg]

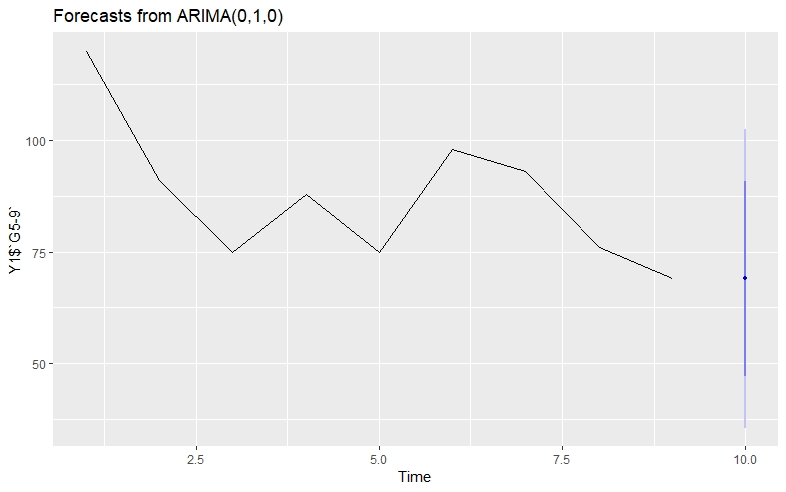

Supplement: Multimedia Appendix 2 [file publichealth_v8i4e36022_app2.zip › Female/F5-9.jpeg]

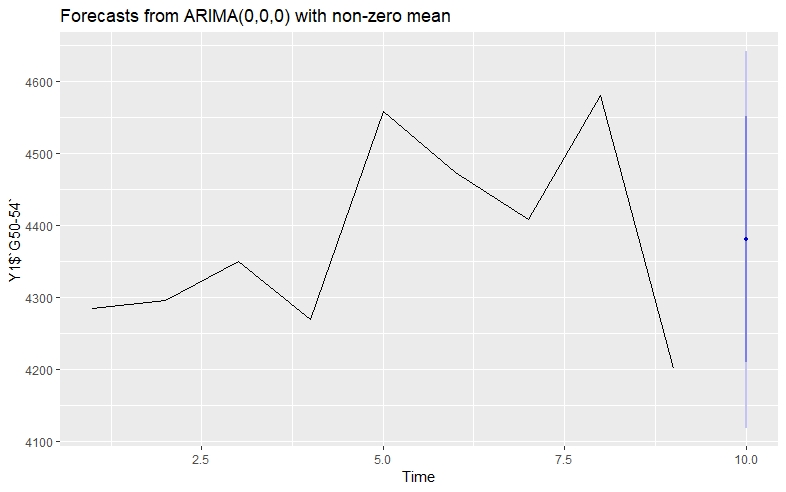

Supplement: Multimedia Appendix 2 [file publichealth_v8i4e36022_app2.zip › Female/F50-54.jpeg]

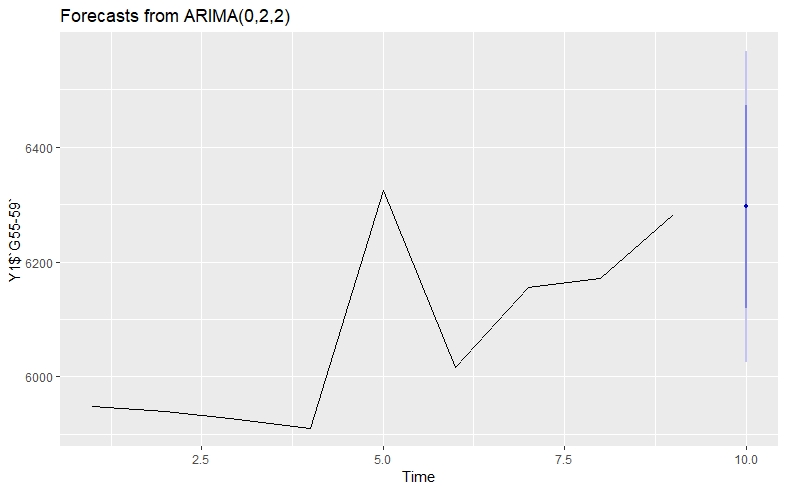

Supplement: Multimedia Appendix 2 [file publichealth_v8i4e36022_app2.zip › Female/F55-59.jpeg]

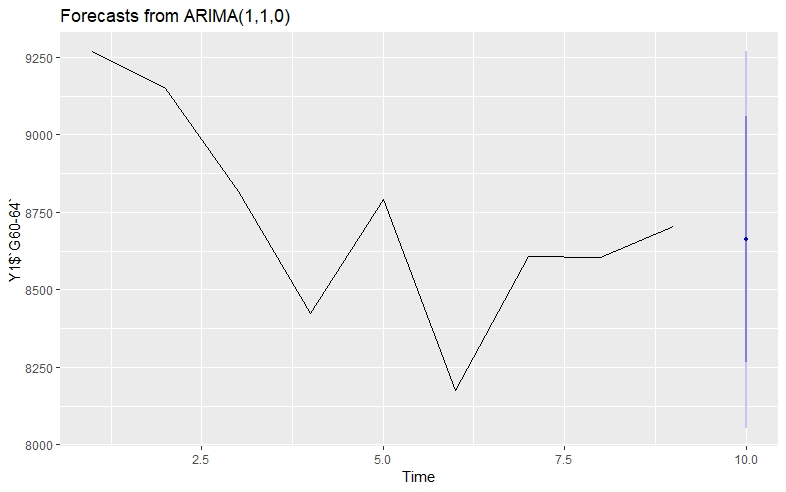

Supplement: Multimedia Appendix 2 [file publichealth_v8i4e36022_app2.zip › Female/F60-64.jpeg]

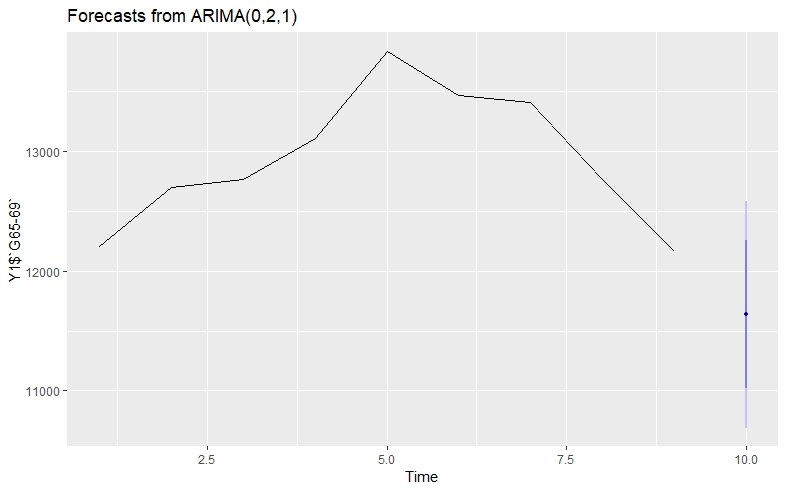

Supplement: Multimedia Appendix 2 [file publichealth_v8i4e36022_app2.zip › Female/F65-69.jpeg]

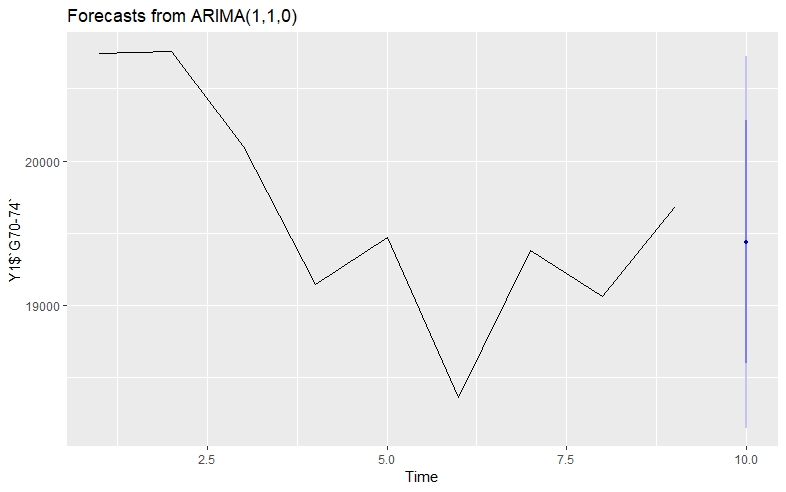

Supplement: Multimedia Appendix 2 [file publichealth_v8i4e36022_app2.zip › Female/F70-74.jpeg]

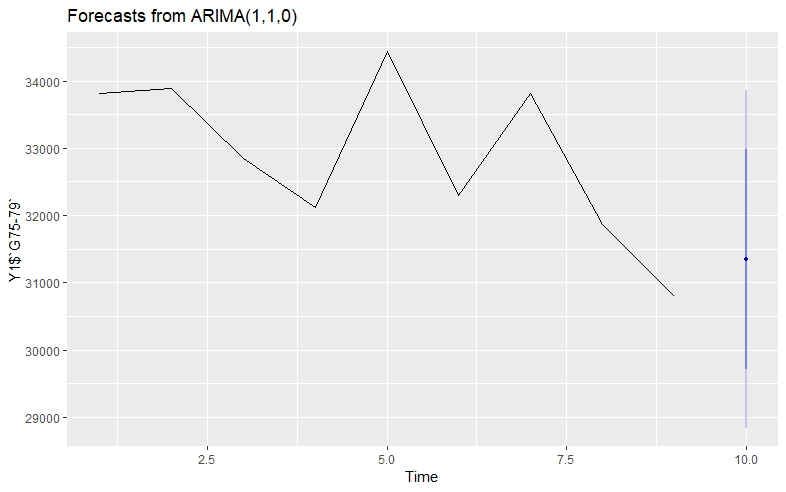

Supplement: Multimedia Appendix 2 [file publichealth_v8i4e36022_app2.zip › Female/F75-79.jpeg]

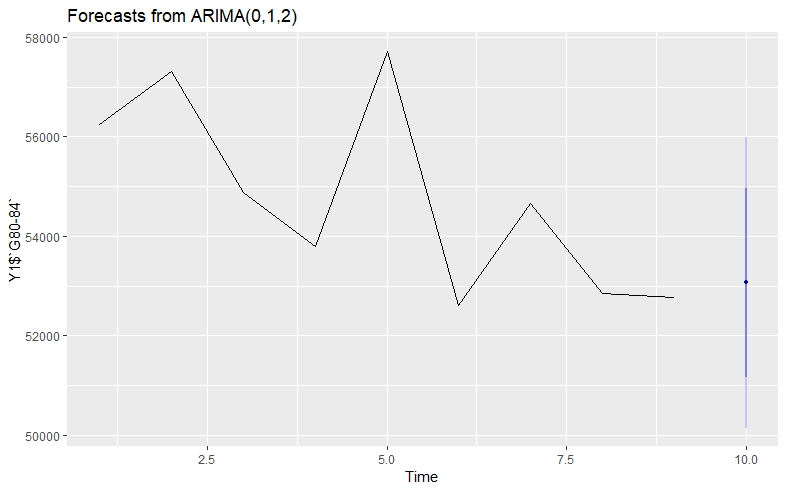

Supplement: Multimedia Appendix 2 [file publichealth_v8i4e36022_app2.zip › Female/F80-84.jpeg]

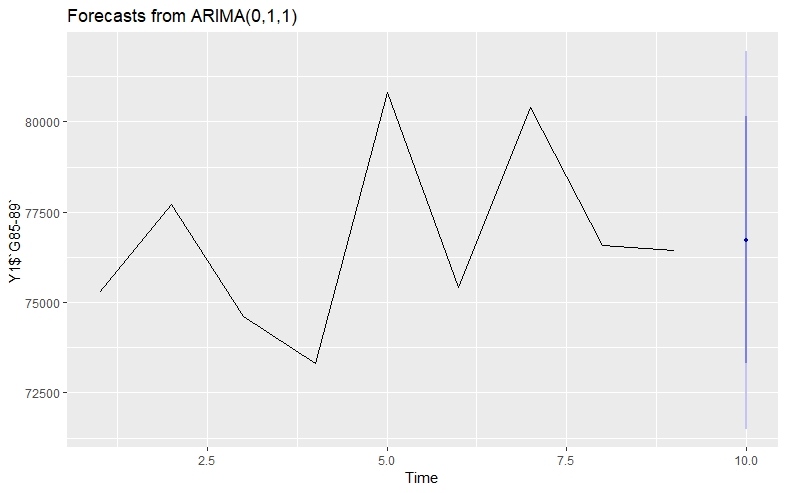

Supplement: Multimedia Appendix 2 [file publichealth_v8i4e36022_app2.zip › Female/F85-89.jpeg]

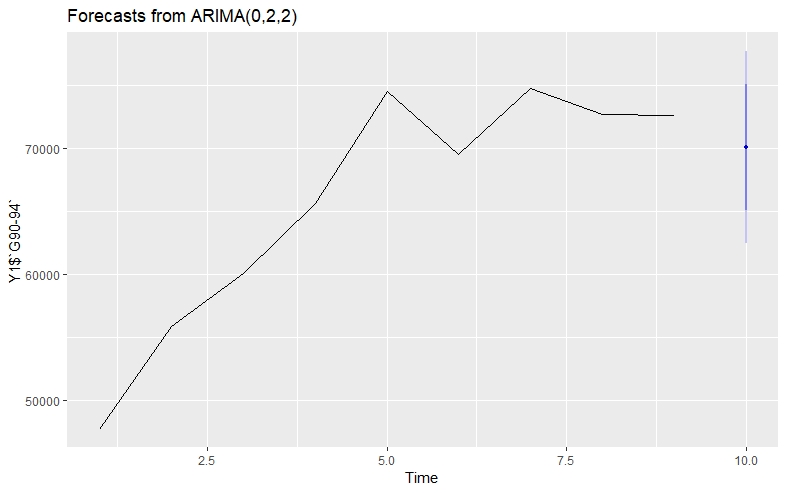

Supplement: Multimedia Appendix 2 [file publichealth_v8i4e36022_app2.zip › Female/F90-94.jpeg]

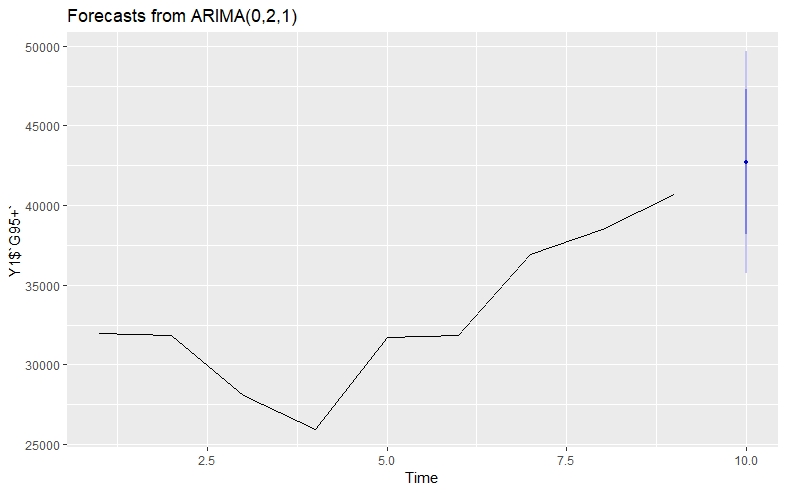

Supplement: Multimedia Appendix 2 [file publichealth_v8i4e36022_app2.zip › Female/F95+.jpeg]

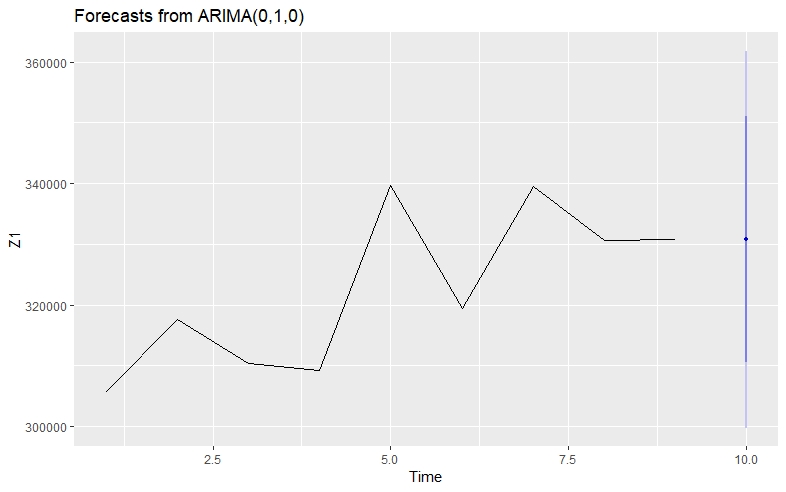

Supplement: Multimedia Appendix 2 [file publichealth_v8i4e36022_app2.zip › Female/FG.jpeg]

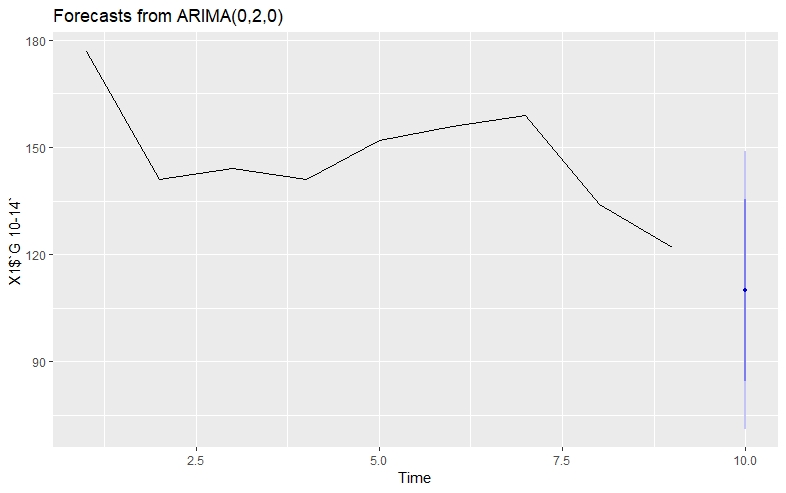

Supplement: Multimedia Appendix 2 [file publichealth_v8i4e36022_app2.zip › Male/M10-14.jpeg]

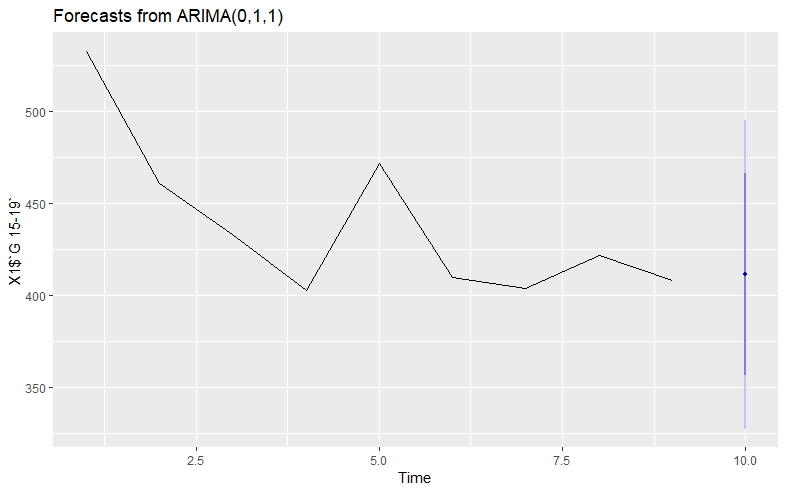

Supplement: Multimedia Appendix 2 [file publichealth_v8i4e36022_app2.zip › Male/M15-19.jpeg]

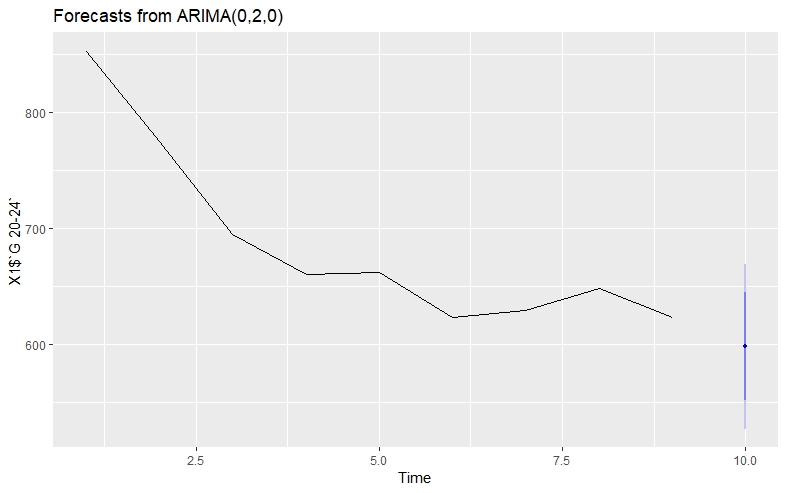

Supplement: Multimedia Appendix 2 [file publichealth_v8i4e36022_app2.zip › Male/M20-24.jpeg]

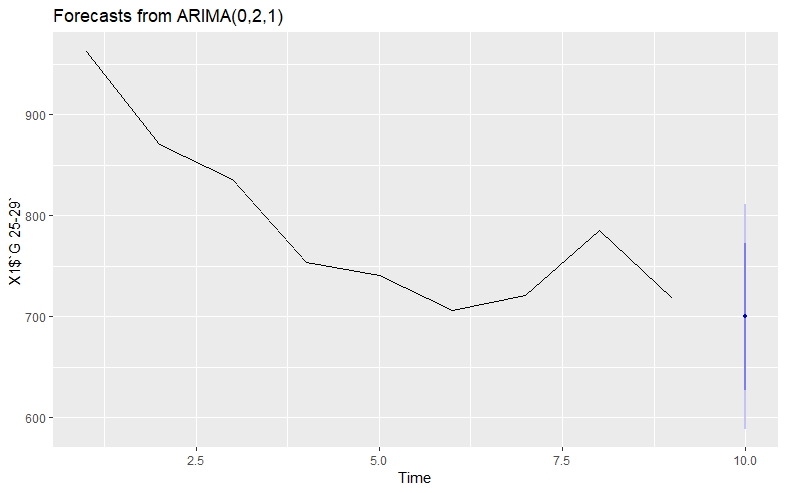

Supplement: Multimedia Appendix 2 [file publichealth_v8i4e36022_app2.zip › Male/M25-29.jpeg]

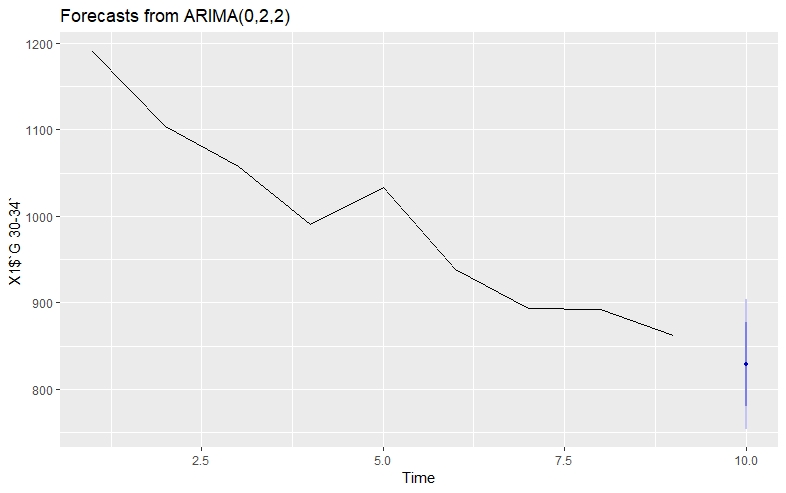

Supplement: Multimedia Appendix 2 [file publichealth_v8i4e36022_app2.zip › Male/M30-34.jpeg]

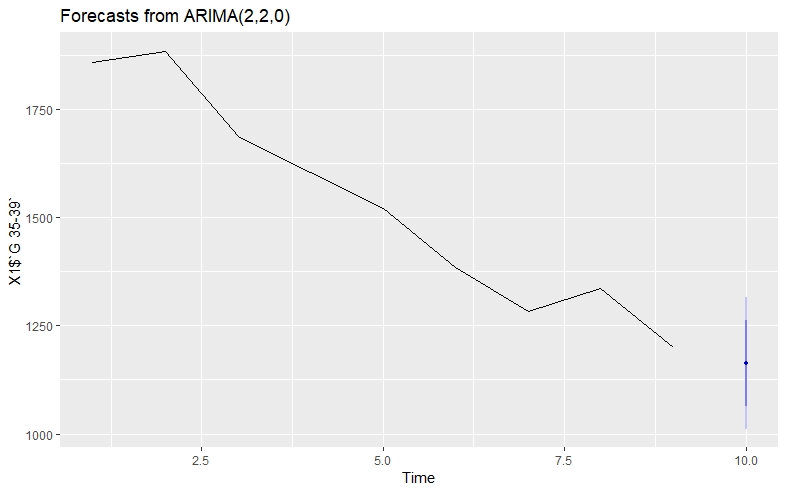

Supplement: Multimedia Appendix 2 [file publichealth_v8i4e36022_app2.zip › Male/M35-39.jpeg]

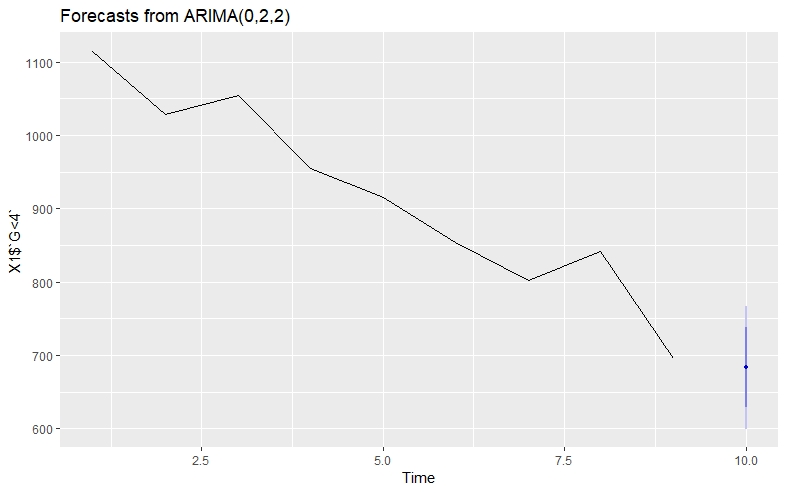

Supplement: Multimedia Appendix 2 [file publichealth_v8i4e36022_app2.zip › Male/M4-.jpeg]

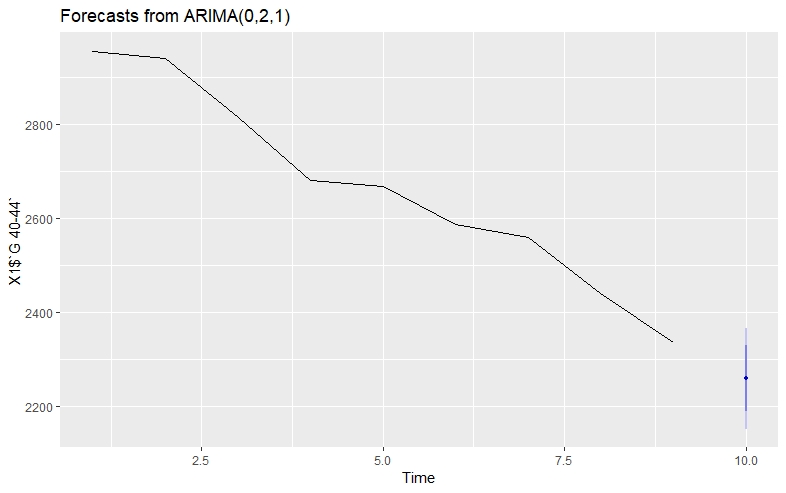

Supplement: Multimedia Appendix 2 [file publichealth_v8i4e36022_app2.zip › Male/M40-44.jpeg]

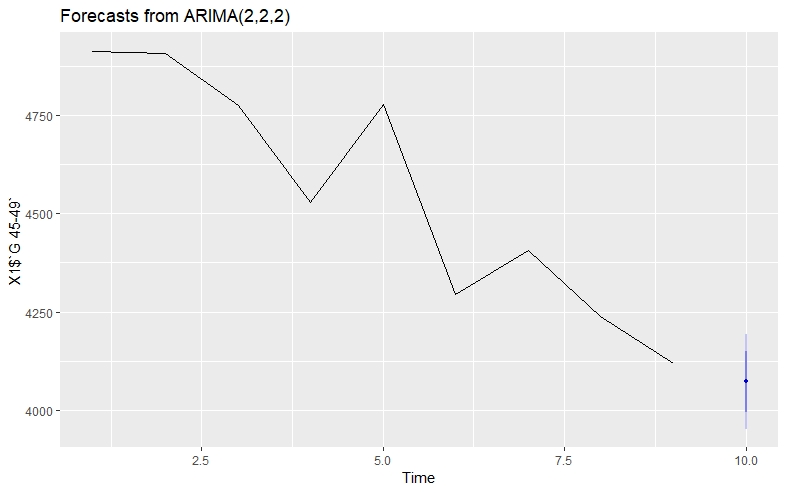

Supplement: Multimedia Appendix 2 [file publichealth_v8i4e36022_app2.zip › Male/M45-49.jpeg]

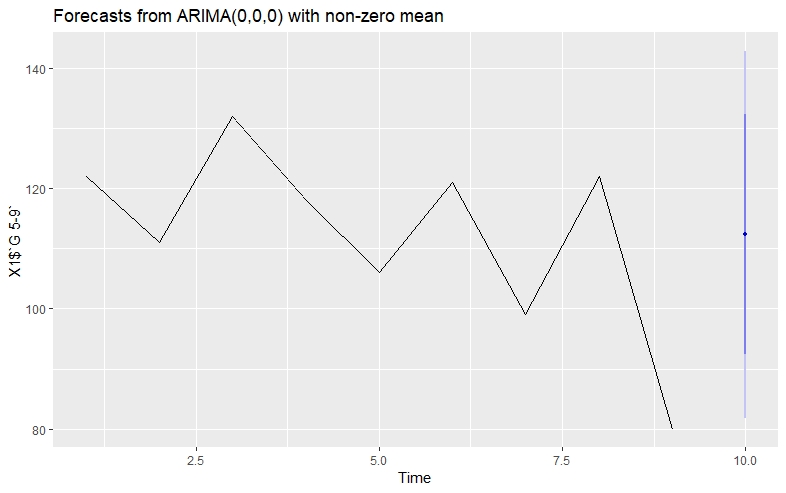

Supplement: Multimedia Appendix 2 [file publichealth_v8i4e36022_app2.zip › Male/M5-9.jpeg]

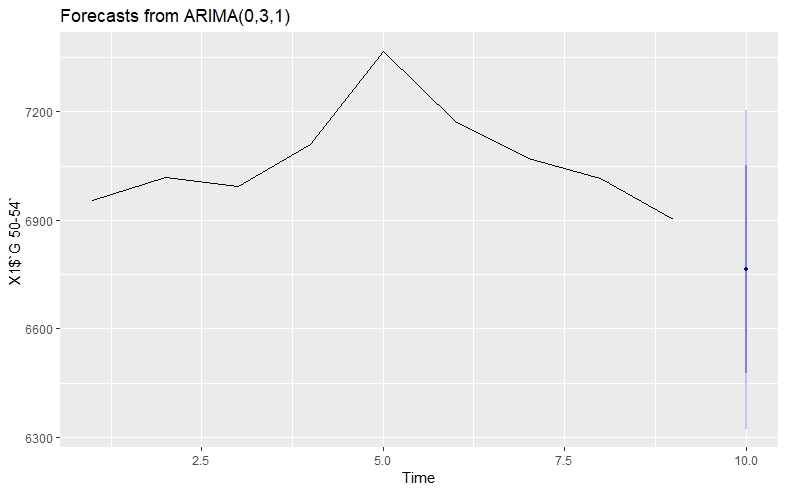

Supplement: Multimedia Appendix 2 [file publichealth_v8i4e36022_app2.zip › Male/M50-54.jpeg]

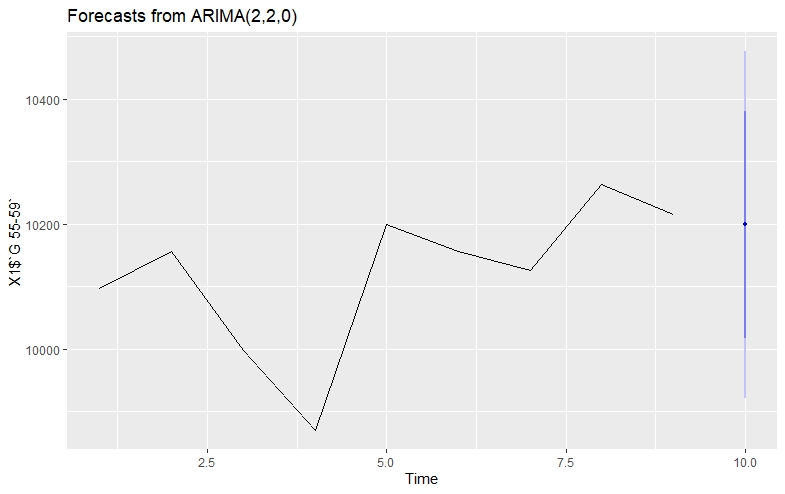

Supplement: Multimedia Appendix 2 [file publichealth_v8i4e36022_app2.zip › Male/M55-59.jpeg]

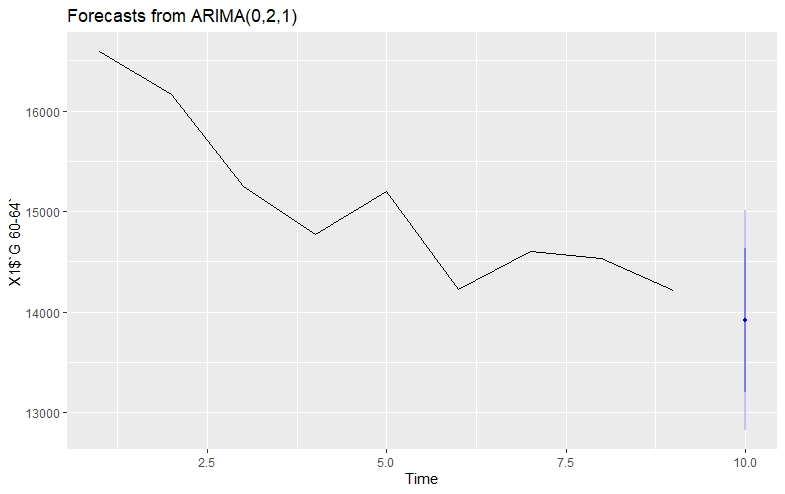

Supplement: Multimedia Appendix 2 [file publichealth_v8i4e36022_app2.zip › Male/M60-64.jpeg]

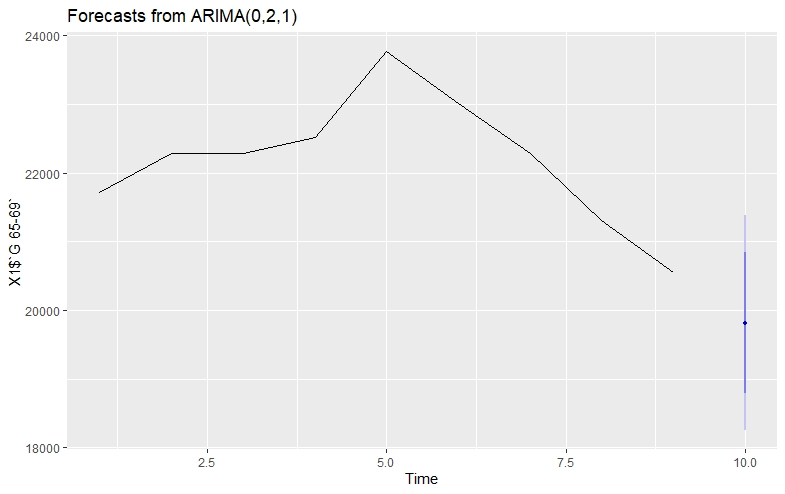

Supplement: Multimedia Appendix 2 [file publichealth_v8i4e36022_app2.zip › Male/M65-69.jpeg]

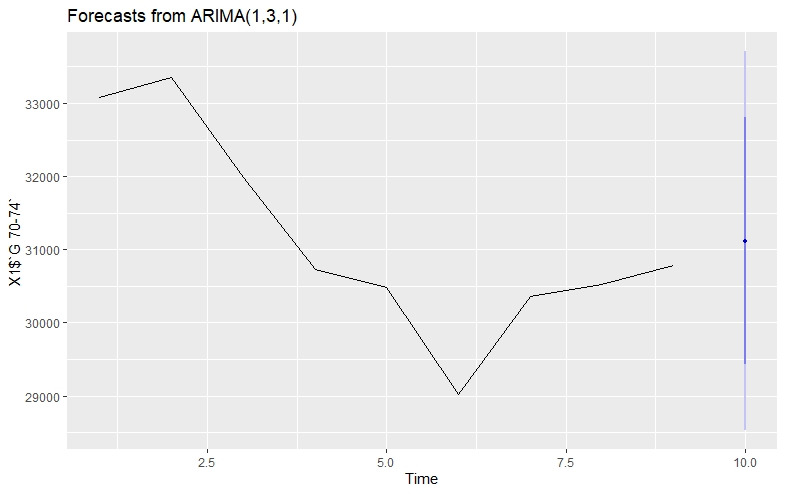

Supplement: Multimedia Appendix 2 [file publichealth_v8i4e36022_app2.zip › Male/M70-74.jpeg]

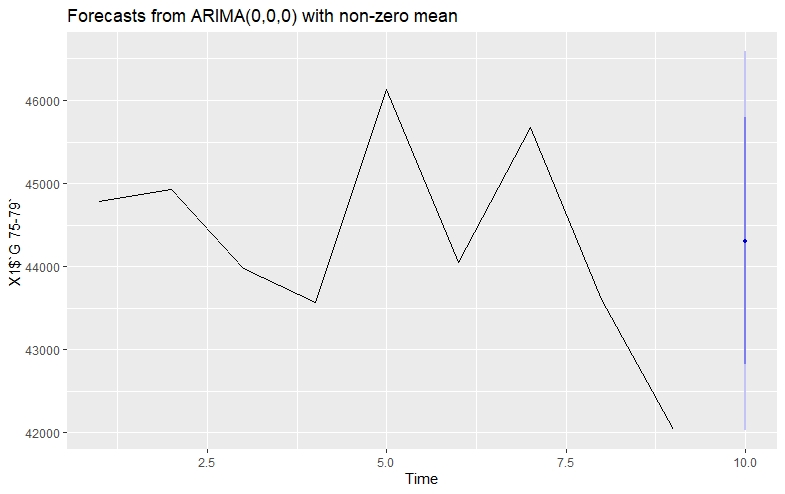

Supplement: Multimedia Appendix 2 [file publichealth_v8i4e36022_app2.zip › Male/M75-79.jpeg]

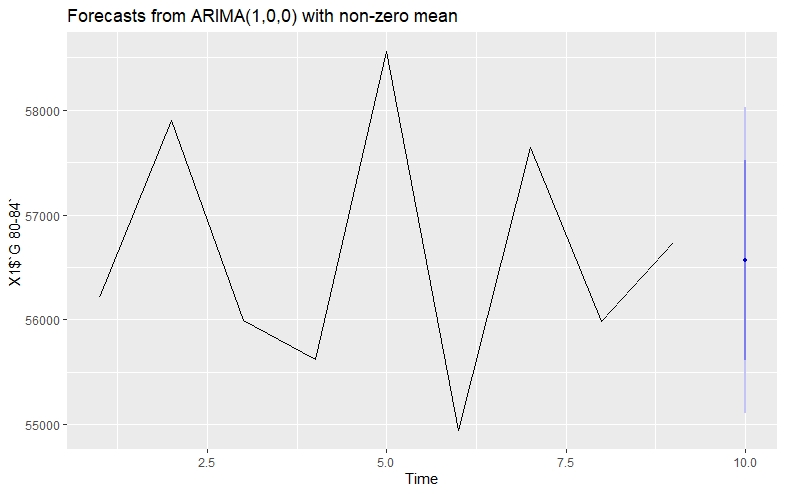

Supplement: Multimedia Appendix 2 [file publichealth_v8i4e36022_app2.zip › Male/M80-84.jpeg]

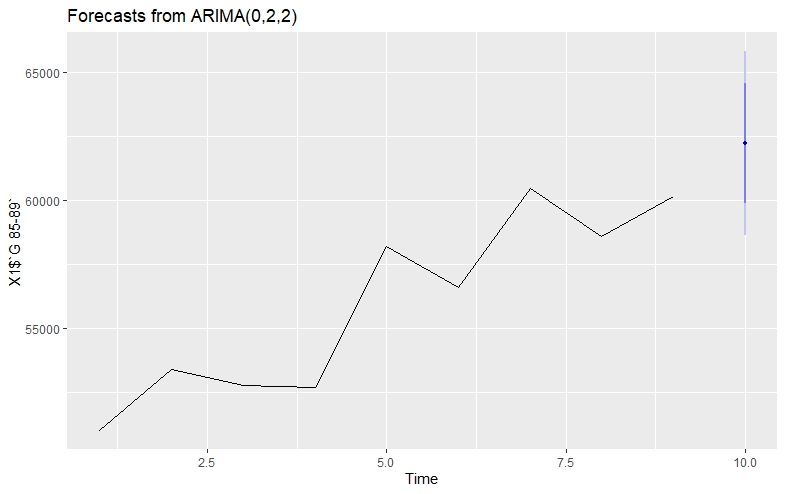

Supplement: Multimedia Appendix 2 [file publichealth_v8i4e36022_app2.zip › Male/M85-89.jpeg]

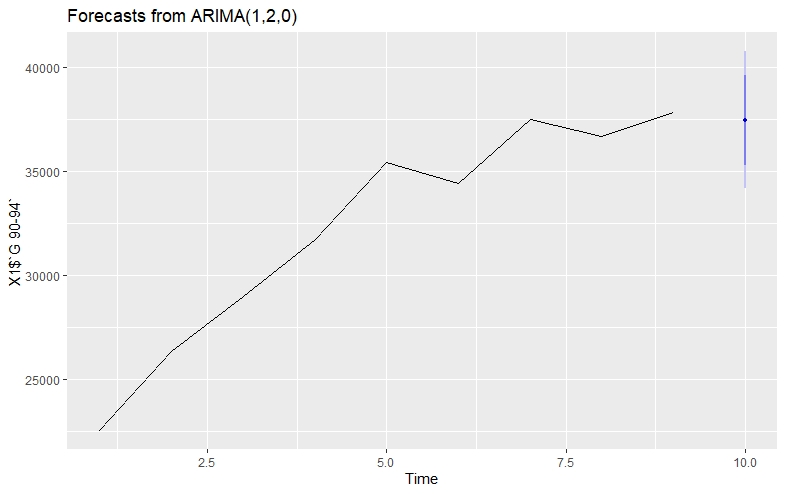

Supplement: Multimedia Appendix 2 [file publichealth_v8i4e36022_app2.zip › Male/M90-94.jpeg]

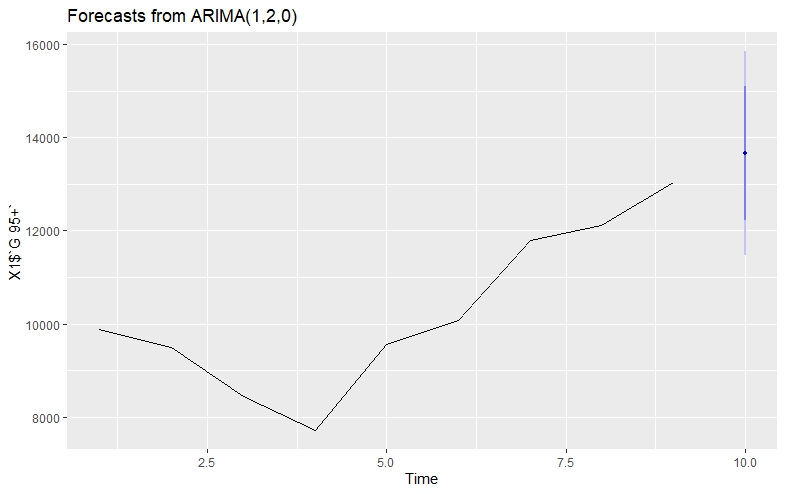

Supplement: Multimedia Appendix 2 [file publichealth_v8i4e36022_app2.zip › Male/M95+.jpeg]

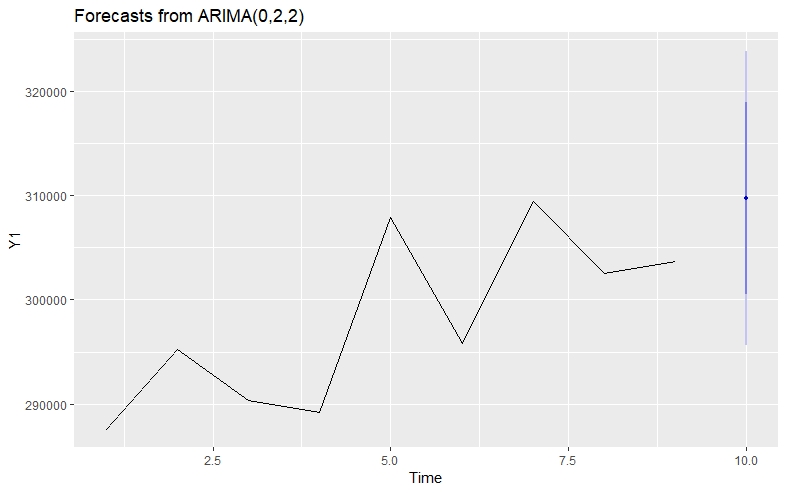

Supplement: Multimedia Appendix 2 [file publichealth_v8i4e36022_app2.zip › Male/MG.jpeg]
